# Expression of distinct maternal and somatic 5.8S, 18S, and 28S rRNA types during zebrafish development

**DOI:** 10.1261/rna.061515.117

**Published:** 2017-08

**Authors:** Mauro D. Locati, Johanna F.B. Pagano, Geneviève Girard, Wim A. Ensink, Marina van Olst, Selina van Leeuwen, Ulrike Nehrdich, Herman P. Spaink, Han Rauwerda, Martijs J. Jonker, Rob J. Dekker, Timo M. Breit

**Affiliations:** 1 RNA Biology and Applied Bioinformatics Research Group, Swammerdam Institute for Life Sciences, Faculty of Science, University of Amsterdam, Amsterdam 1090 GE, the Netherlands; 2 Department of Molecular Cell Biology, Institute of Biology, Leiden University, Gorlaeus Laboratories–Cell Observatorium, Leiden 2333 CE, the Netherlands

**Keywords:** ribosomal RNA, maternal rRNA, embryogenesis, zebrafish, ribosomes

## Abstract

There is mounting evidence that the ribosome is not a static translation machinery, but a cell-specific, adaptive system. Ribosomal variations have mostly been studied at the protein level, even though the essential transcriptional functions are primarily performed by rRNAs. At the RNA level, oocyte-specific 5S rRNAs are long known for *Xenopus.* Recently, we described for zebrafish a similar system in which the sole maternal-type 5S rRNA present in eggs is replaced completely during embryonic development by a somatic-type. Here, we report the discovery of an analogous system for the 45S rDNA elements: 5.8S, 18S, and 28S. The maternal-type 5.8S, 18S, and 28S rRNA sequences differ substantially from those of the somatic-type, plus the maternal-type rRNAs are also replaced by the somatic-type rRNAs during embryogenesis. We discuss the structural and functional implications of the observed sequence differences with respect to the translational functions of the 5.8S, 18S, and 28S rRNA elements. Finally, in silico evidence suggests that expansion segments (ES) in 18S rRNA, previously implicated in ribosome–mRNA interaction, may have a preference for interacting with specific mRNA genes. Taken together, our findings indicate that two distinct types of ribosomes exist in zebrafish during development, each likely conducting the translation machinery in a unique way.

## INTRODUCTION

Ribosomes are large ribonucleoproteins composed of a few noncoding ribosomal RNAs (rRNAs) and many ribosomal proteins (Venema and Tollervey 1999). The rRNAs act as ribozymes by catalyzing the important steps of the amino acid polymerization during protein synthesis (Voorhees and Ramakrishnan 2013). Eukaryotic rRNA consists of four elements: 5S (∼120 nucleotides [nt]), 5.8S (∼160 nt), and 28S (∼4200 nt) in the large 60S subunit (LSU), and 18S (∼1900 nt) in the small 40S subunit (SSU) (Wilson and Cate 2012). The primary roles of the SSU are to orchestrate the binding of the ribosome to mRNA and monitor the complementarity of tRNA and mRNA in translation (Schluenzen et al. 2000; Aitken and Lorsch 2012). The primary functions of the LSU are to link amino acids and terminate translation. The LSU functions are organized in evolutionarily conserved regions of the 28S rRNA: the peptidyl transferase center (PTC), the GTPase-associated center (GAC), and the sarcin–ricin domain (SRD) (Szewczak et al. 1993; Uchiumi and Kominami 1994; Polacek and Mankin 2008).

Whereas 5S rRNA is transcribed from individual genes in tandem repeats by RNA polymerase III (Ciganda and Williams 2011), 18S, 5.8S, and 28S rRNAs originate from the complicated processing of the precursor 45S rRNA, which is derived from a single transcription unit in the genome (45S rDNA) (Henras et al. 2015). These 45S rDNA units are transcribed by RNA polymerase I in the nucleoli and are organized in tandem repeats that occur in several nucleolus organizer regions (NORs) throughout the genome, each containing up to several hundred repeats (Sakai et al. 1995; McStay 2016).

Until recently, scientists often regarded the ribosome as a “constitutive molecular machine” (Barna 2015; Preiss 2016), thereby establishing its image as a kind of steady household organelle with a clear structure and automated functions in which rRNA merely serves as a structural scaffold (Noller 2005). The crystal structure of the ribosome showed that rRNA, besides creating a structural framework for the ribosome, also forms the main features of its functional sites. In fact, the ribosomal functional sites are mostly devoid of ribosomal proteins, which are found mainly at the outer surface of the ribosome and are thought to play primarily a structural and regulatory role (Moore and Steitz 2002b; Noller 2005). Nowadays, similar to histones whose role has been revised from purely structural to regulatory (Campos and Reinberg 2009), the ribosome is being perceived as being much more adaptive than initially proposed (Xue and Barna 2012; Shi and Barna 2015).

Along this line, ribosome variability has been studied mostly at the protein level, where it was shown to play a role in cell- and tissue-specificity, as well as a whole range of crucial biological processes in many organisms including stress response, development, and life cycle (Xue and Barna 2012; Shi and Barna 2015). These studies recently culminated with the assessment of ribosomal heterogeneity across a hundred cell types and tissues in mouse and human (Yadav et al. 2016).

However, rRNA is also a source of ribosomal variability and thereby possibly regulation. First of all, there are differences between species in the so-called expansion segments (ES), which are elements of variable length and sequence of the eukaryotic rRNA when compared to the prokaryotic rRNA core, in both the SSU and LSU (Gerbi 1996; Cannone et al. 2002; Wilson and Cate 2012; Anger et al. 2013). But also within species, heterogeneity of the 45S rDNA was already discovered in the late seventies, which is reflected by the variability of the ribosomal transcription units in both length and sequence (Wellauer et al. 1976; Arnheim and Southern 1977). Further investigations have since shown that the human 28S rRNA has several variants that differ in the sequence of a specific region (Kuo et al. 1996).

Transcending the level of such relatively small rRNA sequence differences is 5S rRNA, where an oocyte-specific variant is known to have substantial sequence differences (Wegnez et al. 1972; Peterson et al. 1980; Korn 1982; Allison et al. 1995). This variant has 20,000 copies in the *Xenopus* genome that are expressed only during oogenesis and early embryogenesis (Wormington and Brown 1983). In contrast, the 5S rRNA variant expressed in somatic tissues, called somatic-type, just consists of 400 rDNA genes. Thus this oocyte-variant probably evolved to enable the production of the enormous amount of 5S rRNA in oocytes equivalent to that of hundreds of thousands of somatic cells (Brown 2004). Recently, we reported similar 5S rRNA variants in zebrafish (Locati et al. 2017), in which a maternal-type 5S r|RNA (2330 genes on chromosome 4) makes up virtually all oocyte 5S rRNA, which is completely replaced during embryogenesis by the somatic-type (12 genes on chromosome 18). 5S rRNA specificity during development has also been discovered in various other species (Komiya et al. 1986; Martins et al. 2002; Martins and Wasko 2004; Danzmann et al. 2007).

The 5S rRNA oocyte types sparked expectation that a similar system might also be present for the other rRNAs, but this has not been observed yet in animals. For example, a study in mouse concluded that there is no difference between the oocyte and embryos as to the expression of 45 rDNA variants (Ihara et al. 2011). However, variation was shown for the parasite *Plasmodium,* in which two distinct 45S rDNA variants are preferentially expressed during the mosquito–host and mammalian–host stage of the parasite life cycle (Gunderson et al. 1987; McCutchan et al. 1988; Rogers et al. 1996; Qi et al. 2015). The functional importance of these two rRNA types is not clear yet (van Spaendonk et al. 2001). More recently, rRNA was shown to also vary at the post-transcriptional level between different cell types in human, which adds to the potential variability of the total ribosome (Krogh et al. 2016).

As the developmental 5S rRNA types in zebrafish have been overlooked for a long time, we investigated the expression of 5.8S, 18S, and 28S rRNAs throughout zebrafish development from egg to adult, with next-generation sequencing. We discovered that the 5.8S, 18S, and 28S rRNAs that accumulate in zebrafish oocytes originate from one specific genomic location and that, similarly to 5S rRNA, these maternal-type rRNAs are gradually replaced by a somatic-type from another genomic location during zebrafish embryogenesis.

All three mature rRNA elements contain substantial sequence differences between their two developmental types, for which we indicate potential effects by examining them in the context of the known folded ribosomal domains. Additionally, we also found indications that the maternal-type 18S rRNA may preferentially interact with mRNA from maternally expressed genes.

## RESULTS AND DISCUSSION

### 45S rDNA types in the zebrafish genome

It is known that the 45S rDNA transcription units occur in tandem repeats in eukaryotic genomes, with a copy number up to 400 in humans (Henras et al. 2015). Also, 45S rDNA variants have been observed in several organisms (Kuo et al. 1996; Rogers et al. 1996; Ihara et al. 2011). To study potential 5.8S, 18S, and 28S rRNA variations in zebrafish, we made an inventory of the zebrafish 45S rDNA units present in the current zebrafish genome assembly (GRCz10). All rDNA units in the zebrafish genome assembly encompass, with one exception, only one complete rDNA unit. Altogether, we identified five complete and three partial 45S rDNA units on zebrafish chromosomes 4 and 5, as well as three complete and one partial clone that were not part of the genome assembly (Supplemental Table S1A). All 45S rDNA units that were either partial or not in the genome assembly showed high similarity (≥99%) to the five complete 45S rDNA units present on chromosomes 4 or 5. Hence, we focused our analysis on these complete genomic 45S rDNA units. Comparison revealed substantial difference in sequence and size of the transcribed spacers and rRNA elements (Supplemental Table S1; Supplemental File S1). Of the five genomic rDNA units, three in close proximity on chromosome 4 are very similar, while the other two are quite different (similarity down to just 86%) in their rRNA elements (Supplemental Table S1). Hence, there appears to be three different 45S rDNA types. At the same time, we are well aware that correct annotation of repetitive sequences, especially long ones, is a known challenge that can lead to misassembly and incorrect copy-number estimation (Treangen and Salzberg 2012). Therefore, given the available genome data, we were able to identify three distinct types of 45S rDNA units based on their sequences. These 45S rDNA types each have their specific locus on two chromosomes, while their copy numbers remain unknown.

**FIGURE 1.**
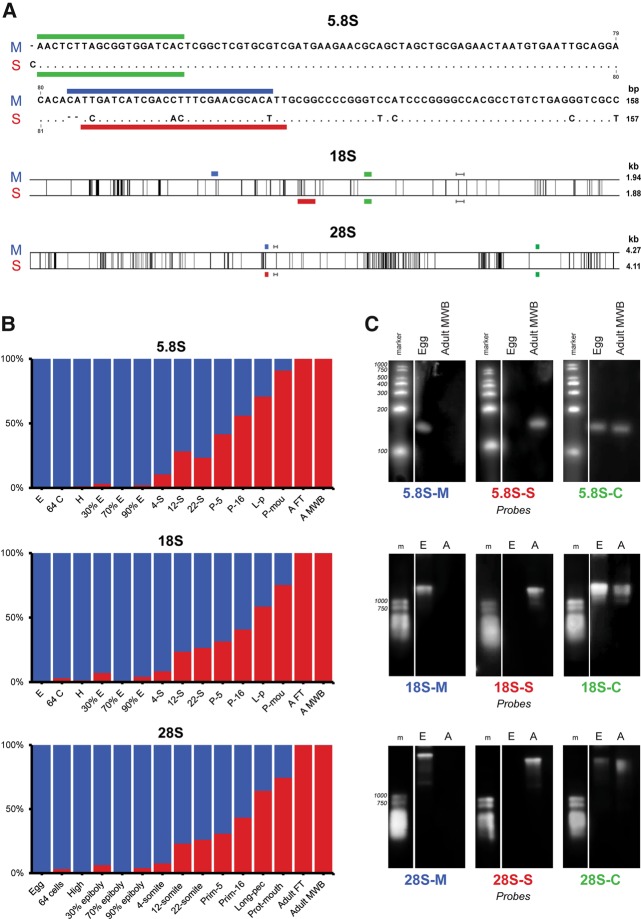
Expression of the maternal- and somatic-type rRNA types in zebrafish development. (*A*) Alignments of the maternal-type (M) and somatic-type (S) rRNAs. In the 5.8S rRNA alignment, identical nucleotides are indicated as dots, gaps as dashes. In the 18S and 28S rRNA alignments, the black *vertical* lines indicate the locations where maternal- and somatic-type differ (due to the scale, not all differences are visible; for a complete alignment check Supplemental File S1). The probes used for Northern blotting (panel *C*) are indicated with colored lines (green: -C, specific to a region common to both types; blue: -M, specific for maternal-type; red: -S, specific for somatic-type). The dark gray *horizontal* lines show the position of the regions used to discriminate between the three types of rRNA (cf. main text and Supplemental Table S1). (*B*) Expression of the maternal-type (blue) and somatic-type (red) rRNAs indicated by percentage of total 5.8S, 18S, and 28S rRNA sequencing reads, respectively. Prot-mouth, protruding-mouth; Adult FT, adult-female tail; Adult MWB, adult-male whole-body. (*C*) Northern blot analyses with total RNA from zebrafish eggs and adult-male whole-body tissue (Adult MWB) and probes as indicated in *A*. Each panel contains lanes from the same gel, but with adjusted brightness and contrast for visual clarity.

Usually not all 45S rDNA variants present in a genome are expressed (Kuo et al. 1996), and therefore we analyzed the expression of the 18S, 5.8S, and 28S rRNAs in a range of zebrafish tissues to identify whether all three 45S rDNA variant types are transcribed. For 5.8S rRNA, we applied an adapted small-RNA-seq approach and for 18S as well as 28S rRNA a rRNA-seq approach (see Materials and Methods) on the following: an egg pool (51 M and 48 M reads, respectively), an embryonic time series (49 M reads and 79 M reads), whole-body adult-male samples (40 M and 40 M reads), and female-adult tail samples (7.7 M and 9.8 M reads) in order to include somatic female tissues without ovaries and developing oocytes. Analysis of the rRNA-seq reads using small 18S and 28S rRNA specific sequences (26 nt long) that discriminate the three rRNA variant types (Fig. 1A; Supplemental Table S2), revealed that only two of the three 45S rDNA variant types appeared to be abundantly transcribed, while the type with three 45S rDNA units on chromosome 4 had virtually no expression (Supplemental Table S2).

### Developmental stage-specific expression of two 45S rDNA types

Even with the limited analysis using only small type-specific 45S rDNA sequences, it became immediately clear that the two expressed 45S rDNA types showed stage-specific expression during development (Supplemental Table S2). Much like we discovered before with zebrafish 5S rRNA (Locati et al. 2017), there is one 5.8S, 18S, and 28S rRNA type that is virtually absent in eggs, but makes up almost 100% of the rRNA in adult tissue (Fig. 1B; Supplemental Table S3). In contrast, the other 5.8S, 18S, 28S rRNA type makes up almost 100% of the rRNA in eggs and is virtually absent in adult tissue. Similarly to 5S rRNA, we named these two types somatic-type (45S-S) and maternal-type (45S-M) rRNA, respectively (Fig. 1A). Northern blot analysis with probes specific for 5.8S, 18S, and 28S rRNA (Fig. 1A) clearly confirmed the rRNA sequencing results (Fig. 1C). Analysis of 12 intermediate embryonic-development stages showed that there is a noticeable increase of somatic-type rRNA as early as the 64 cell stage and a steady increase starting at epiboly (Fig. 1B; Supplemental Table S3). This is in line with the observations that somatic rRNAs are first transcribed during gastrulation in amphibian embryogenesis (Brown and Caston 1962; Brown and Littna 1964). Although we and others have found 5S rRNA variation in various organisms (Wegnez et al. 1972; Komiya et al. 1986; Martins and Wasko 2004; Locati et al. 2017), to our knowledge there has never been a report on differentially expressed 5.8S, 18S, and 28S rRNA types in any animal. Taken together, these data suggest that there are maternal-type and somatic-type ribosomes.

Intriguingly, the zebrafish maternal-type 45S rDNA locus is located on the long arm of the chromosome 4, where the maternal-type 5S gene cluster is found (Locati et al. 2017). This genomic region is also known for its abundance of tRNA genes, scarcity of protein-coding genes, and extensive heterochromatin (≈transcriptional silencing) in somatic cells (Howe et al. 2013). It is worth mentioning an important difference between the maternal-type 5S rDNA and the maternal-type 45S rDNA: Whereas the enormous amount of rRNA in oocytes is achieved for 5S genes by over a thousand gene copies in the genome, for the production of 5.8S, 18S, and 28S rRNA there is an additional DNA amplification step needed, in which extrachromosomal circles are produced (Rochaix et al. 1974). With the discovery of a maternal-type 5.8S, 18S, and 28S rRNA, we logically assumed that we also identified the locus that is amplified during oogenesis and present as extrachromosomal circles.

To verify this, with the knowledge that the amplified copies are still present in mature eggs (Thomas et al. 1977), we analyzed the copy number of maternal- and somatic-type 45S rDNA units with qPCR, in both single zebrafish eggs and adult tissue. Our results showed that egg genomic DNA contains ∼1000 times more copies of maternal-type 45S rDNA compared to genomic DNA from somatic cells. Moreover, the somatic-type 45S rDNA turned out not to be amplified in eggs (Supplemental Fig. S1). This proves that indeed only the maternal-type rRNA locus is specifically amplified during oogenesis.

Given the assumption that maternal-type rRNA transcription is stopped in a mature oocyte (Newport and Kirschner 1982), the replacement of maternal rRNA must be achieved by a combination of somatic-type rRNA transcription and maternal-type rRNA degradation. This would however require a turnover of maternal-type ribosomes higher than the reported ribosome half-life of 4–6 d in rat liver and brain (Stoykova et al. 1983) or 9–31 d in *Xenopus* oocytes (Brown and Gurdon 1964), as virtually all maternal-type rRNA has disappeared at about 3 d post-fertilization.

### Structural and functional implications of the LSU rRNA types

**FIGURE 2.**
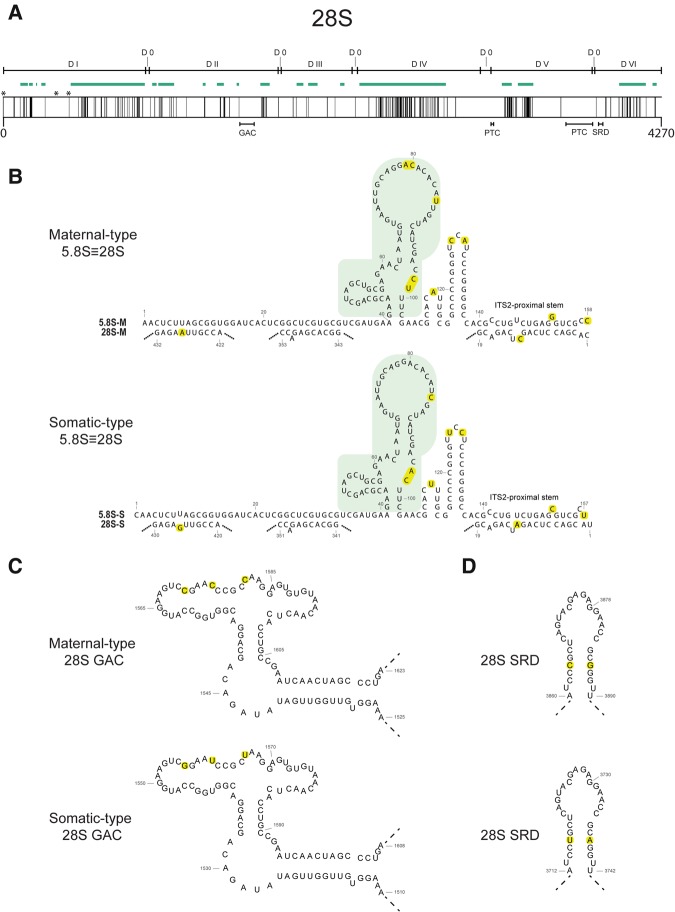
Differences between zebrafish maternal- and somatic-type 5.8S and 28S rRNA. (*A*) Plot with the differences between maternal- and somatic-type 28S rRNA in relation to the structural domains (“D”: Domain), expansion segments (ES, green lines [Anger et al. 2013]) and functional domains (GAC, GTPase-associated center; PTC, peptidyl transferase center; SRD, sarcin–ricin domain). The black *vertical* lines indicates the spots where the maternal- and somatic-type differ (due to the scale, not all differences are visible). Asterisks indicate the 28S rRNA regions that interact with 5.8S rRNA. (*B*) The putative secondary structures for maternal- and somatic-type 5.8S rRNA and their interactions with the equivalent 28S rRNAs are shown (Petrov et al. 2014). The region that undergoes the conformational switch and interacts with ribosome-dissociating factors is highlighted in light green (Graifer et al. 2005). The nucleotides that differ between the two types are marked (yellow). (*C*) Putative secondary structures for maternal- and somatic-type GTPase-associated center (GAC). The nucleotides that differ between the two types are marked (yellow). (*D*) Putative secondary structures for maternal- and somatic-type sarcin–ricin domain (SRD). The nucleotides that differ between the two types are marked (yellow).

The primary functions of the ribosomal large subunit (LSU) are to covalently link amino acids via peptide bonds through peptidyl transferase activity and the termination of translation (Polacek and Mankin 2008). These functions are mainly performed by 5.8S, 28S, and 5S rRNA components of the LSU supported by the associated riboproteins (Moore and Steitz 2002a). For 5.8S rRNA, the sequence differences between maternal- and somatic-type are primarily present in the central and terminal regions (Figs. 1A, 2B). This is in contrast to the sequence differences between maternal- and somatic-type 5S rRNA, which are mainly present in the 5′ half (Locati et al. 2017).

In the LSU, 5.8S rRNA is bound to 28S via three base-paired elements (Fig. 2A,B). In the proximal element, the 3′ end of the 5.8S together with the 5′ end of the 28S form the ITS2-proximal stem. This structure is highly conserved within the three eukaryote kingdoms of plants, fungi, and animals (Keller et al. 2009), and the ITS2-proximal stem (helix 10) is essential in processing 5.8S and 28S (Peculis and Greer 1998). Between maternal and somatic types, only three nucleotides differ in the ITSs-proximal stem: two in 5.8S and another in 28S rRNA. Noticeably, the difference in 5.8S rRNA was a nucleotide with no pairing nucleotide, and the difference in 28S rRNA was a nucleotide next to a nonpairing nucleotide (Fig. 2B). A thorough study in yeast (Côté and Peculis 2001) has shown that the structure of the ITS2-proximal stem (helix 10) is critical for the formation of mature rRNA, while the sequence specificity plays a lesser role. This seems also to be true in zebrafish: The sequence differences in the maternal-type ITS2-proximal stem (helix 10) are not sufficient to block pre-rRNA maturation, as ample mature 5.8S and 28S were detected in eggs; however, it might affect the efficiency and/or timing of the pre-rRNA processing. Within the two other base-paired elements, only one 28S rRNA nucleotide was shown to be different between the two rRNA types (Fig. 2B). Hence, this situation is different from the 5.8S–28S rRNA combinations that are present in the developmental 45S rDNA types in *Plasmodium falciparum*, where specific combinations are directed by covariations (Gunderson et al. 1987; McCutchan et al. 1988; Waters et al. 1989).

All other sequence differences between maternal- and somatic-type 5.8S rRNA also seem to be in non-double-stranded nucleotides and conspicuously at the top of two stem–loops. Hence the sequence differences between the 5.8S rRNA types seem less involved with the direct 5.8S and 28S rRNA base-paired binding, but likely more with protein binding and conformation. For instance, the 5.8S rRNA central region is thought to undergo a conformational switch after disconnection of all ligands from the ribosome, facilitating the dissociation of the two ribosomal subunits (Graifer et al. 2005). Interestingly, the maternal-type 5.8S has an insertion of AC at positions 79–80; this is a region that in the free ribosomes has a peculiar stem–loop structure that promotes binding with ribosome-dissociating factors (Fig. 2B).

Sequence differences between maternal- and somatic-type 28S rRNA have been found in all seven 28S rRNA structural domains, albeit with variant occurrences from 0.6% to a staggering 20.8% (Supplemental Tables S4, S5). The dispersed domain zero (Petrov et al. 2013) is thus the most conserved between the 28S rRNA types. The majority of differences seem to coincide with the known eukaryotic expansion segments (ESs) (Fig. 2A; Leffers and Andersen 1993). ESs exhibit a significant degree of variability between species in length and sequence, plus their size and number of helical branches seem to progressively increase in higher eukaryotes (Michot and Bachellerie 1987). The role of the 28S ESs in ribosomal function is still not clear, but it has recently been shown that they are important in specific steps of the ribosome biogenesis in yeast (Ramesh and Woolford 2016). The many differences we observe between the ESs of maternal- and somatic-type 28S will undoubtedly have an effect on assembly and functioning of the ribosomal LSU.

On the other hand, there are evolutionarily conserved regions of the 28S rRNA with known specific functions, such as the peptidyl transferase center (PTC), the GTPase-associated center (GAC), and the sarcin–ricin domain (SRD) (Fig. 2A; Doris et al. 2015). The PTC catalyzes the two principal chemical reactions of protein synthesis, peptide bond formation, and peptide release (Polacek and Mankin 2008); the GAC binds elongation factors and activates their GTPase activity (Uchiumi and Kominami 1992); and the SRD anchors the elongation factor on the ribosome during mRNA–tRNA translocation (Szewczak et al. 1993; Shi et al. 2012). It is worth noting that the somatic-type 28S rRNA sequences of these three functional centers have a 100% identity with phylogenetically distant species such as human (Supplemental Table S1). This makes it even more striking that there are several differences in these areas between zebrafish maternal- and somatic-type 28S rRNA. In the PTC there is just 1 nt difference: U3780C. But even a single difference in such a conserved region is worth noticing, knowing that in the prokaryote PTC-equivalent, individual nucleotides have specific roles in the translation process and some mutations result in a lethal phenotype (Beringer 2008; Polacek and Mankin 2008; Long et al. 2009, 2010; Yang et al. 2009; Chirkova et al. 2010).

Binding of ribosomal factors to the GAC is fundamental for ribosome function, and the 3-nt differences in the maternal-type GAC (Fig. 2C), again present in non-stem sequences, could have a role in modulating these interactions as is known in both prokaryotes (Xu et al. 2002) and eukaryotes (Uchiumi and Kominami 1992, 1994).

The SRD displays two differences between the rRNA types in a stem region (Fig. 2D). The fact that it is a U–A base pair in the somatic-type and a G–C base pair in the maternal-type makes these differences fall in the category of covariations, bringing additional support to the importance of this stem structure for the role of SRD.

It is obvious that the many differences present between maternal- and somatic-type 5.8S and 28S rRNA sequences deserve more attention as to their meaning in the translation machinery, but potentially also for their role at the DNA level, as was true for the differences between maternal- and somatic-type 5S rRNA genes that resulted in different retrotransposon target sites (Locati et al. 2017).

### Structural and functional implications of the SSU rRNA types

**FIGURE 3.**
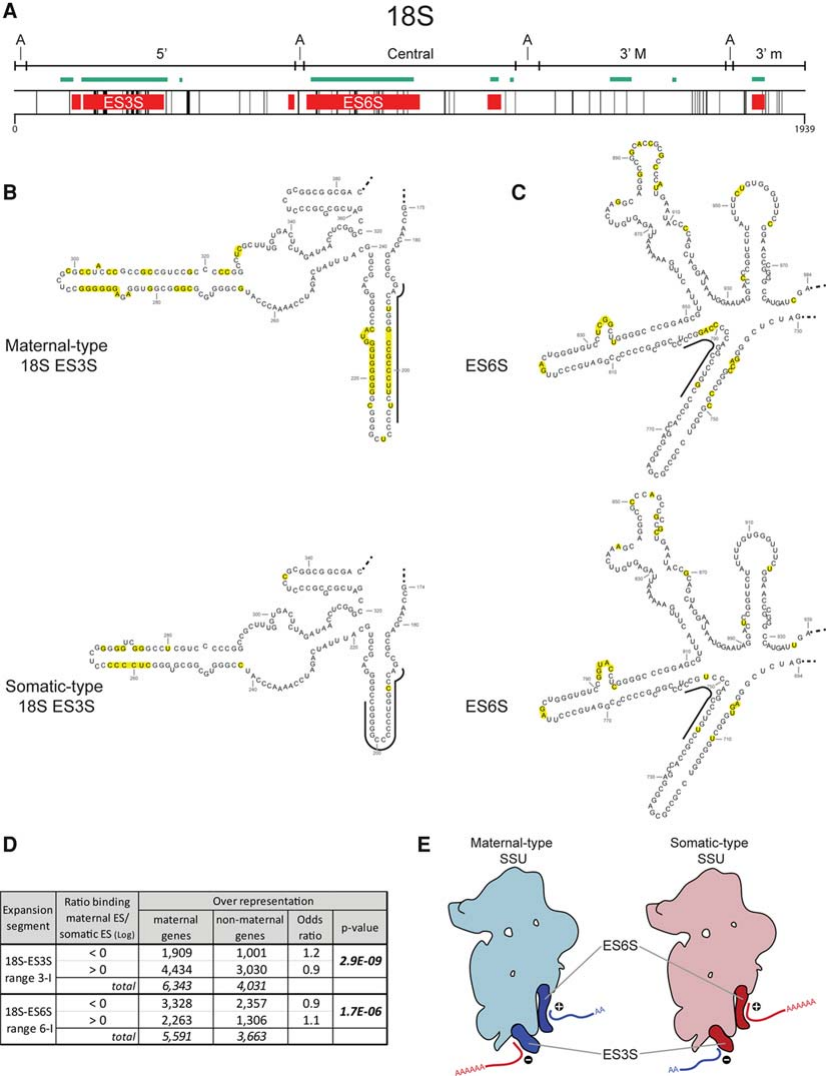
Differences between zebrafish maternal- and somatic-type 18S rRNA. (*A*) Schematic representation of the differences between maternal- and somatic-type 18S rRNA in correlation with the structural domains (*A*, Domain A; 5′, Domain 5′; Central, Central Domain; 3′ M, 3′ Major domain; 3′ m, 3′ minor domain), expansion segments (ES, green lines [Anger et al. 2013]) and sticky regions (red boxes [Pánek et al. 2013]). The black vertical lines indicates the spots where the maternal- and somatic-type 18S rRNA sequences differ (due to the scale, not all differences are visible). (*B*) Putative secondary structures for maternal- and somatic-type sticky regions corresponding to ES3S. The nucleotides that differ between the two types are marked (yellow). Thick black lines indicate the regions (“range 3-I,” nucleotides: maternal-type 188–207; somatic-type 187–206) analyzed in panel *D*. (*C*) Putative secondary structures for maternal- and somatic-type sticky regions corresponding to ES6Ss. The nucleotides that differ between the two types are marked (yellow). Thick black lines indicate the regions (“range 6-I,” nucleotides: maternal-type 776–797; somatic-type 738–756) analyzed in panel *D*. (*D*) Table presenting the overrepresentation of maternal genes versus nonmaternal genes with respect to the binding of each gene transcript to 18S-ES3S and 18S-ES6S. The mentioned ranges correspond with the ranges indicated in panels *B* and *C*. (*E*) Graphical representation of the binding of maternal (blue line) and somatic (red line) mRNA transcripts to the indicated sticky regions of the SSU types. The + and − indicate the translation stimulating and repressing interactions, respectively, at the indicated ES sites.

The primary functions of the ribosomal small subunit (SSU) are to initiate mRNA engagement and monitor the complementarity of tRNA and mRNA in translation (Schluenzen et al. 2000; Aitken and Lorsch 2012). 18S rRNA is the main component of the ribosomal small subunit (SSU) with four distinct peripheral domains: 5′, central, 3′ major, and 3′ minor (Fig. 3A; Supplemental Table S5), which form distinct 3D structures (Henras et al. 2015). Recently, a fifth central domain has been defined: domain A, which is quite conserved between different organisms (Gulen et al. 2016). This domain is the structural and functional core of the 18S rRNA that connects and orientates all the four peripheral domains, which may explain why it, much like its counterpart domain zero in 28S rRNA, contains the lowest percentage of nucleotide variation (2.9%) between the zebrafish 18S rRNA types (Supplemental Table S4; Supplemental Fig. S2). The other four peripheral domains contain many sequence differences, with the 5′ domain containing an impressive 10.3% (Supplemental Table S4). Taking the 3′ minor domain as an example, eight of the nine altered nucleotides in the central part of the long stem are paired and two of these four pairs are covariations (Supplemental Fig. S2), also further supporting the importance of this stem domain. This is in line with the observation that point mutations in the structural analog of *E. coli* (Jemiolo et al. 1985) showed altered growth. Conversely, the second smaller stem close to the 3′ end has been proven to be essential for translational initiation in *E. coli*: A 2-nt mutation in this region was lethal. In line with this observation, no difference between the zebrafish maternal and somatic-type 18S rRNA was found in this stem.

The “ribosome filter hypothesis” (Mauro and Edelman 2002, 2007; Mauro and Matsuda 2016) proposes that the interactions between ribosomes and mRNA can influence translation, giving the eukaryotic ribosome the ability to “filter” which mRNA will be translated. One way eukaryotic mRNAs can interact with ribosomes is via sequence complementarity to rRNA, in particular 18S rRNA (Tranque et al. 1998; Alkemar and Nygård 2004). The interactions by base-pairing of mRNAs to the 18S rRNA are located within the translated (Tranque et al. 1998; Martin et al. 2016) as well as in the untranslated regions (UTRs) of the mRNAs (Barendt et al. 2012; Pánek et al. 2013), and it seems that they can both favor (Barendt et al. 2012) and inhibit translation (Verrier and Jean-Jean 2000). Recently, 18S “sticky regions” were defined as complementary to parts of the mRNA 5′ UTR, being very conserved in evolution and located in the solvent-exposed areas of the SSU surface (Pánek et al. 2013). The fact that there are two different ribosome types in one organism, which are expressed in combination with distinct gene sets (maternal versus somatic), provides an opportunity to study the possible interaction between rRNA and mRNA. For this, we traced the zebrafish sticky regions in the maternal- and somatic-type 18S rRNA and found that they show quite some sequence variation (Fig. 3A–C). Comparing the “stickiness” of a large 18S rRNA expansion segment 6 (ES6S) (Fig. 3A) for 5′ UTRs of mRNAs showed a clear difference between the somatic- and maternal-type 18S rRNAs (Supplemental Fig. S3), as the distribution of their ratio was lower than one. This means that different mRNAs have dissimilar affinity for the maternal- and somatic-type 18S ES6S. This was underlined by the analysis of another ES, ES3S, which also showed a markedly shifted, higher than one, maternal/somatic-type ratio distribution (Supplemental Fig. S3).

To substantiate the difference between mRNA binding to the maternal- and somatic-type 18S-rRNAs, we zoomed in on particular stretches of these ESs that contained clear nucleotide differences (Supplemental Fig. S3) and combined these data with known maternally expressed mRNAs. Even with this relative naïve approach, we were able to quite convincingly show that for ES6S (determined by range I, Fig. 3C), the maternal-type 18S rRNA “matches” better with maternally expressed mRNAs, whereas somatic-type 18S rRNA matches better with maternally nonexpressed mRNAs (Fig. 3D). Unexpectedly, for ES3S (range I, Fig. 3B) this was shown to be reversed (Fig. 3D). As in the initial phase of zebrafish embryogenesis, only maternal mRNAs and maternal-type ribosomes are present, and their matching is expected to facilitate the overall translation process. Hence, we propose that the 18S rRNA ES6S may be a site to selectively recruit maternal mRNA that need to be translated during embryogenesis (Fig. 3E), while vice versa, the SSU ES3S might be a site to selectively recruit mRNAs to prevent their translation by sequestering (Fig. 3E). In this way, the ES3S and ES6S may effectively enhance the selective properties of the SSU in an elegant manner, thus promoting the discriminating operation of the maternal- and somatic-type ribosomes.

### Conclusion

In recent years there has been mounting evidence that the ribosome is part of an adaptive transcription machinery in living cells. This has broadly been shown by the variability in ribosomal proteins, especially during embryonic development (Kondrashov et al. 2011; Xue and Barna 2012). Our study adds to this by showing that in zebrafish there exist at least two ribosomal systems that comprise quite distinct versions of 5.8S, 18S, and 28S rRNA, besides the previously reported 5S rRNA. Parallel to 5S rDNA, there are distinct maternal-type and somatic-type 45S rDNA transcription units, with their own chromosomal location, unique sequence, as well as a specific expression pattern. Maternal-type 5.8S, 18S, and 28S rRNA are expressed during oogenesis and replaced by somatic-type during embryogenesis. Hence, there are maternal-type ribosomes and somatic-type ribosomes in zebrafish. Despite the fact that *Xenopus* has maternal- and somatic-type 5S rRNA, we could not find evidence of any maternal-type 18S, 5.8S, and 28S rRNA (results not shown).

Besides the inevitable functional consequences associated with the many sequence differences present between these two rRNA types, these also seem to have an effect on the ribosome–mRNA interaction. It appears that there may be 18S rRNA sites that either promote the translation process via interaction between ribosomes and mRNAs of the same type, or sites that hamper it via sequestering interaction between ribosomes and mRNA of different types. The fact that in this study we provide two ribosomal types in one organism will support the growing awareness of “specialized ribosomes” and could help the identification and elucidation of the many ribosomal functions that are hidden in this enormously complex and intriguing transcription machinery.

## MATERIALS AND METHODS

### Biological materials

#### Zebrafish

Adult zebrafish (strain ABTL) were handled in compliance with local animal welfare regulations and maintained according to standard protocols (http://zfin.org). The breeding of adult fish was approved by the local animal welfare committee (DEC) of the University of Leiden, the Netherlands. All protocols adhered to the international guidelines specified by the EU Animal Protection Directive 86/609/EEC. Unfertilized eggs (oocytes) were collected by squeezing the abdomen of three spawning females and further stored as three corresponding egg pools. Whole-body male-adult zebrafish samples, female-adult tail samples, and egg pools were flash-frozen in liquid nitrogen and stored at −80°C. Before freezing, fish were put under anesthesia using 0.02% buffered 3-aminobenzoic acid ethyl ester (Tricaine).

#### Zebrafish embryonic time course

Zebrafish embryos were collected immediately after fertilization, maintained at 28.5°C, and staged using standard morphological criteria (Kimmel et al. 1995). One embryo was collected at 12 embryonic development points: 1, 64 cells (2 hpf); 2, high stage (3.3 hpf); 3, 30% epiboly stage (4.7 hpf); 4, 70% epiboly stage (7 hpf); 5, 90% epiboly stage (9 hpf); 6, 4-somite stage (11.3 hpf); 7, 12-somite stage (15 hpf); 8, 22-somite stage (20 hpf); 9, prim-5 stage (24 hpf); 10, prim-16 (31 hpf); 11, long-pec stage (48 hpf); 12, protruding-mouth stage (72 hpf). After collection, the embryos were snap-frozen in liquid nitrogen and stored at −80°C. In order to maintain a uniform genetic background, all embryos were collected from the same batch of fish stock.

### RNA isolation

Five zebrafish samples (three egg pools further pooled together, one whole-body male-adult, and one female-adult tail) and 12 embryos were pulverized to a fine powder under liquid nitrogen using a mortar and pestle. TRIzol reagent (Thermo Fisher Scientific) was added and the manufacturer's instructions were followed to obtain the aqueous phase, which was subsequently removed and combined with 1.5 volumes of ethanol. This mixture (containing total RNA including small RNAs <200 nt) was further purified using the E.Z.N.A. MicroElute RNA Clean Up Kit (Omega Bio-Tek). Next, the large RNA fraction (>200 nt) was obtained from the total RNA using the mirVana miRNA Isolation Kit (Thermo Fisher Scientific). At the same time, the small RNA fraction (<200 nt) was purified from the flow-through by adding ethanol to a final concentration of 65% and loading this onto an E.Z.N.A. MicroElute spin column. The column was washed one round each with buffers RWT and RPE (QIAGEN), and 80% ethanol. RNA concentration was measured on a NanoDrop ND-2000 (Thermo Fisher Scientific), and RNA integrity was examined using the 2200 TapeStation System with Agilent RNA ScreenTapes (Agilent Technologies).

### Next-generation sequencing

Barcoded RNA-seq and small-RNA-seq libraries were generated according to the manufacturer's protocols using the Ion Total RNA-Seq Kit v2 and the Ion Xpress RNA-Seq Barcoding Kit (Thermo Fisher Scientific), except for the small RNA libraries in which both cDNA and amplified cDNA were subjected to a single step purification using a higher ethanol volume to increase the selected fragment sizes (up to 200 nt). The size distribution and yield of the barcoded libraries were assessed using the 2200 TapeStation System with Agilent D1000 ScreenTapes (Agilent Technologies). Sequencing templates were prepared on an Ion Chef System using the Ion PI Hi-Q Chef Kit (Thermo Fisher Scientific). Sequencing was performed on an Ion Proton System using Ion PI Chips v3 (Thermo Fisher Scientific) according to the instructions of the manufacturer.

### qPCR

#### Primer design

Specific PCR primers were designed for the zebrafish maternal-type 5.8S rRNA gene, the somatic-type 5.8S rRNA gene, and p53 as the internal reference gene (Supplemental Table S6).

#### Real-time PCR

Genomic DNA was isolated from zebrafish eggs (*n* = 3) and a whole adult-male zebrafish (*n* = 1). The entire DNA content of each egg was used as the template for copy-number determination. For the adult male, 0.35, 3.5, and 35 ng DNA were used as templates. PCR reactions were prepared according to the manufacturer's instructions, containing Platinum SYBR Green qPCR SuperMix-UDG (Thermo Fisher Scientific), 0.2 μM of each primer, and a genomic DNA template. No-template controls were performed for each primer combination. Real-time PCR was performed on a 7300 Applied Biosystems Thermocycler instrument (Applied Biosystems) using the following program: 50°C for 2 min, 95°C for 2 min, followed by 45 cycles of 95°C for 15 sec and 60°C for 30 sec. The results were analyzed using SDS software v1.4.0 (Applied Biosystems).

### Northern blotting

#### Probe design

Three different DNA 5′-biotinylated probes were designed for each of the three rRNA elements: one common to both maternal-type and somatic-type (-C), one specific for maternal-type (-M), and one specific for somatic-type (-S) (Supplemental Table S6). The probes were ordered from Integrated DNA Technologies or Exiqon, upon arrival immediately rehydrated with LowTE (10 mM Tris, pH 8, and 0.1 mM EDTA) to 100 µM and stored at −20°C.

#### Electrophoresis

For 5.8S, 1 µg zebrafish egg or whole-body male-adult RNA and 1 µL 0.1× biotinylated sRNA Ladder (Kerafast) were mixed with Novex TBE-Urea Sample Buffer and heated at 70°C for 3 min. The samples were loaded on a Novex TBE-Urea (7 M) gel, 6% acrylamide (Thermo Fisher Scientific) following the manufacturer's protocol and run at 70 V for 105 min. For 18S and 28S, 1 µg zebrafish egg or whole-body male-adult RNA and 1 µL 0.1× biotinylated sRNA Ladder (Kerafast) were mixed with a denaturating buffer (500 µL formamide, 100 µL 10× MOPS, 100 µL 80% glycerol–0.2% bromophenol blue, 120 µL formaldehyde, 2 µL 10 mg/mL EtBr) and loaded in a 0.8% Agarose gel with formaldehyde and run at 100 V for 90 min.

#### Blotting and detection

After electrophoresis (Supplemental Fig. S4), the gel was soaked in 20× SSC for 10 min. RNA was subsequently transferred to an Amersham Hybond-N+ (GE Healthcare) membrane by capillary blotting, overnight. The RNA was cross-linked to the membrane by exposure to short-wave UV light (254 nm) for 1 min. The membrane was immediately prehybridized with ULTRAhyb Ultrasensitive Hybridization Buffer (Thermo Fisher Scientific) for 2 h at 55°C. Five picomoles of the selected probe was hybridized overnight to the prehybridized blot at 55°C. Blots were washed twice 5 min in 2× SSC, 0.1% SDS at 55°C, and twice 15 min in 0.1× SSC, 0.1% SDS at 55°C. Detection was performed using the Chemiluminescent Nucleic Acid Detection Module Kit (Thermo Fisher Scientific) following the manufacturer's protocol. Pictures were made with an Odyssey Fc (LI-COR Biosciences) adjusting brightness and contrast when needed.

### Bioinformatics analyses

#### rDNA annotation

The *H. sapiens* GenBank sequences for 18S, 5.8S, and 28S rRNA (U13369.1) were aligned to the *Danio rerio* genome (GRCz10) using BLAST. A genomic locus was recognized as a zebrafish 45S rDNA unit, if it contained all three rRNA elements. 45S rDNA units were annotated “Complete” if all rRNA elements had a minimal sequence length (18S = 1.8 Kb; 5.8S = 150 bp; 28S = 4.0 Kb); “Partial” if just one of the rRNAs did not meet the required minimum length; or not, if more than one rRNA element was incomplete. The same procedure was used to recognize 45S rDNA units in unplaced genomic clones. At the 5′ of 18S and the 3′ of 28S rDNA, 1 kb genomic sequences were selected as putative external transcribed spacers (ETSs).

#### rRNA annotation and secondary structures

The start and end positions of each rRNA (5.8S, 18S, and 28S) were determined using the most abundant rRNA reads from the NGS sequencing. Expansion segments were adapted from *Homo sapiens* as described in Anger et al. (2013). The 18S conserved “sticky regions” (four major regions that are in ESs 3S, 6S, 7S, 12S, and two minor in front of the ESs 3S and 6S) were adapted from Pánek et al. (2013). Zebrafish rRNAs secondary structures and structural domains were modeled after the 3D ribosome structure of *Homo sapiens* (http://apollo.chemistry.gatech.edu/RibosomeGallery) (Petrov et al. 2014).

#### Finding the expressed 45S rDNA variants

Subsequences specific for 45S-M (Maternal), 45S-U (Undetermined), and 45S-S (Somatic) rDNA were selected for 18S and 28S (Supplemental Table S2B) and searched in the fastq files of each NGS sample.

#### Mapping NGS reads to the rRNA types

Reads longer than 100 nt from all small-RNA-seq samples were mapped against maternal- and somatic-type 5.8S rRNA sequences using Bowtie2 (Langmead and Salzberg 2012) with the following settings: -np to 0, - - score-min to L, -0, -0.3, - -rdg and - -rfg to 1,6 in order to limit the maximal amount of mismatches to 5%. SAMtools v1.2 (Li et al. 2009) was used to convert the alignment to the BAM file format and to retrieve the mapped read counts. Reads longer than 25 nt from all rRNA-seq samples were mapped against maternal- and somatic-type 18S and 28S rRNA sequences, similarly to that of the small-RNA-seq samples.

#### 18S sticky regions analysis

The affinity of the 18S ES3S and ES6S regions, as well as the affinity of specific ranges within the 18S ES3S and ES6S (cf. Fig. 3B–D), was determined by calculating each possible ungapped and unidirectional alignment of 5 nt or longer of the ribosomal stretch with all available 5′ UTRs of zebrafish RNA transcripts using the BLAST package version 2.2.29. The 5′ UTRs were retrieved from Ensembl BioMart and, in order to be able to compare our results with the transcriptomics set of Rauwerda et al. (2016), Ensembl release version 79 was used. For each 5′ UTR the number of aligning nucleotides with both the maternal and somatic rRNA variant was determined. Per gene, the counts were averaged and the ratio of the counts on the maternal- and somatic-type (ms-ratio) was calculated; ms-ratios in maternally expressed and nonexpressed genes were calculated using the set of expressed and nonexpressed genes from Rauwerda et al. (2016). Overrepresentation of maternally expressed genes in the sets with ms-ratios smaller or larger than one was calculated with a Fisher's exact test implemented in the R stats package, while excluding genes with a ms-ratio of exactly one.

## DATA DEPOSITION

All sequencing data are accessible through the BioProject database under the project accession number PRJNA347637 (www.ncbi.nlm.nih.gov/bioproject).

## Supplementary Material

Supplemental Material
