# Supplementary material for: Expression of distinct maternal and somatic 5.8S, 18S, and 28S rRNA types during zebrafish development
Source: RNA. 2017 Aug;23(8):1188–99. doi: 10.1261/rna.061515.117 (PMC5513064; doi:10.1261/rna.061515.117)
Supplement: Supplemental Material [file supp_23_8_1188__index.html]

Expression of distinct maternal and somatic 5.8S, 18S, and 28S rRNA types during zebrafish development — Supplemental Material 

# Expression of distinct maternal and somatic 5.8S, 18S, and 28S rRNA types during zebrafish development

## Supplemental Material

**Files in this Data Supplement:**

- Supplemental\_Fig\_S1.pdf
- Supplemental\_Fig\_S2.pdf
- Supplemental\_Fig\_S3.pdf
- Supplemental\_Fig\_S4.pdf
- Supplemental\_File\_S1.pdf
- Supplemental\_Legends.docx
- Supplemental\_Table\_S1.xlsx
- Supplemental\_Table\_S2.xlsx
- Supplemental\_Table\_S3.xlsx
- Supplemental\_Table\_S4.xlsx
- Supplemental\_Table\_S5.xlsx
- Supplemental\_Table\_S6.xlsx
