# Supplementary material for: Expression of distinct maternal and somatic 5.8S, 18S, and 28S rRNA types during zebrafish development
Source: RNA. 2017 Aug;23(8):1188–99. doi: 10.1261/rna.061515.117 (PMC5513064; doi:10.1261/rna.061515.117)
Supplement: Supplemental Material [file supp_061515.117_Supplemental_Fig_S1.pdf]

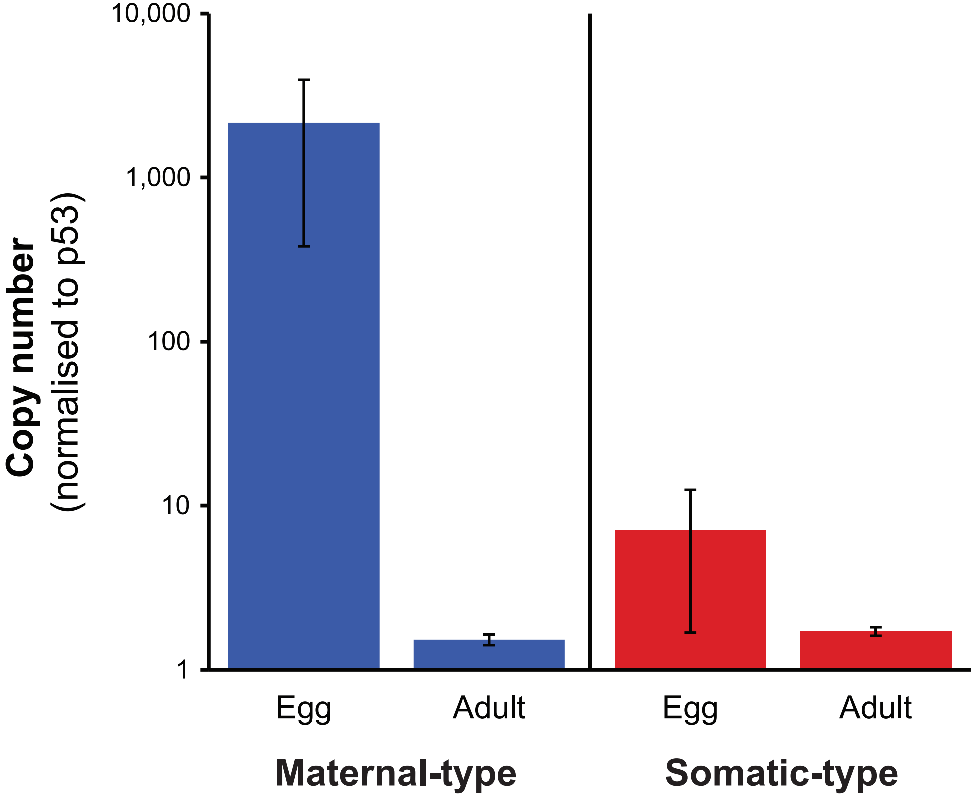

Supplementary Figure S1
Relative copy number of maternal- and somatic-type 45S rDNA in single eggs and adult-male whole-body, calculated as $2^{\Delta Ct}$ to the reference gene (p53). Data represent means ± Standard Error.
