# Supplementary material for: Expression of distinct maternal and somatic 5.8S, 18S, and 28S rRNA types during zebrafish development
Source: RNA. 2017 Aug;23(8):1188–99. doi: 10.1261/rna.061515.117 (PMC5513064; doi:10.1261/rna.061515.117)
Supplement: Supplemental Material [file supp_061515.117_Supplemental_Fig_S2.pdf]

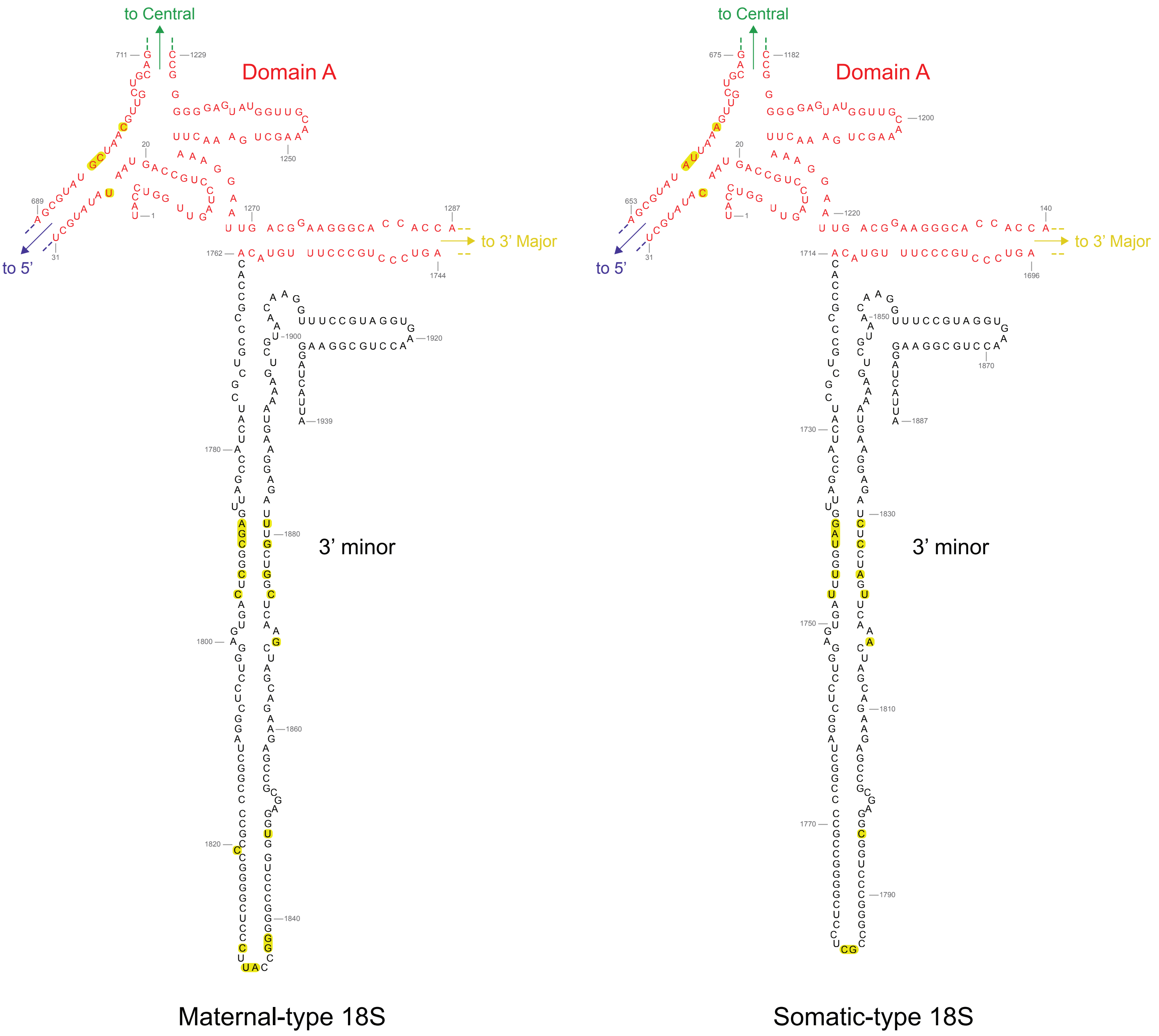

Supplementary Figure S2

The putative secondary structures for maternal- and somatic-type 18S rRNA Domain A (red) and 3' minor (black). The nucleotides that differ between the two types are marked (yellow).
