# Supplementary material for: Expression of distinct maternal and somatic 5.8S, 18S, and 28S rRNA types during zebrafish development
Source: RNA. 2017 Aug;23(8):1188–99. doi: 10.1261/rna.061515.117 (PMC5513064; doi:10.1261/rna.061515.117)
Supplement: Supplemental Material [file supp_061515.117_Supplemental_Fig_S3.pdf]

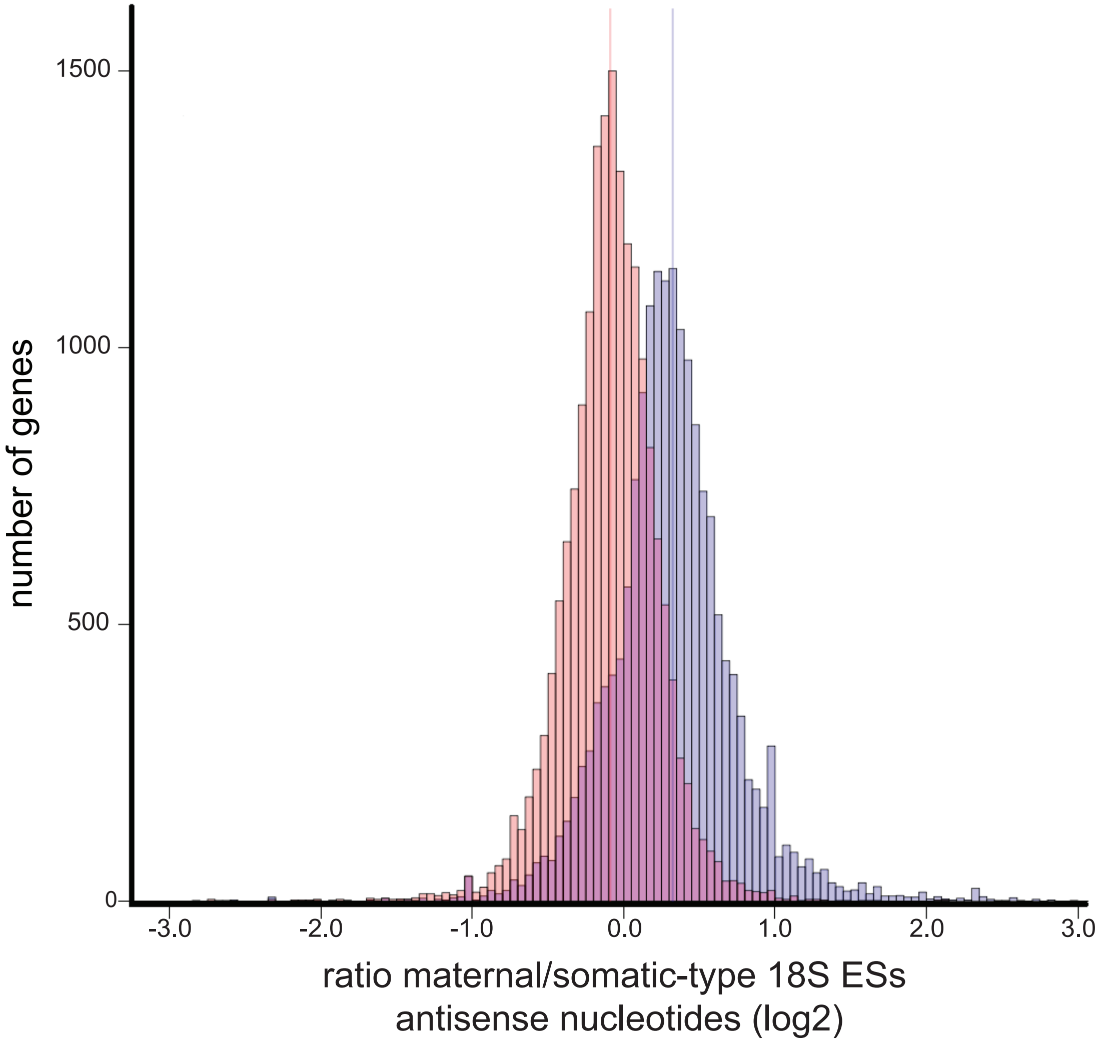

Supplementary Figure S3
The distributions of the per gene (non-equal) ratio of maternal/somatic-type 18S ESs nucleotides that are antisense to the 5'UTR of mRNA (red: ES6; blue: ES3).
