# Supplementary material for: Expression of distinct maternal and somatic 5.8S, 18S, and 28S rRNA types during zebrafish development
Source: RNA. 2017 Aug;23(8):1188–99. doi: 10.1261/rna.061515.117 (PMC5513064; doi:10.1261/rna.061515.117)
Supplement: Supplemental Material [file supp_061515.117_Supplemental_Fig_S4.pdf]

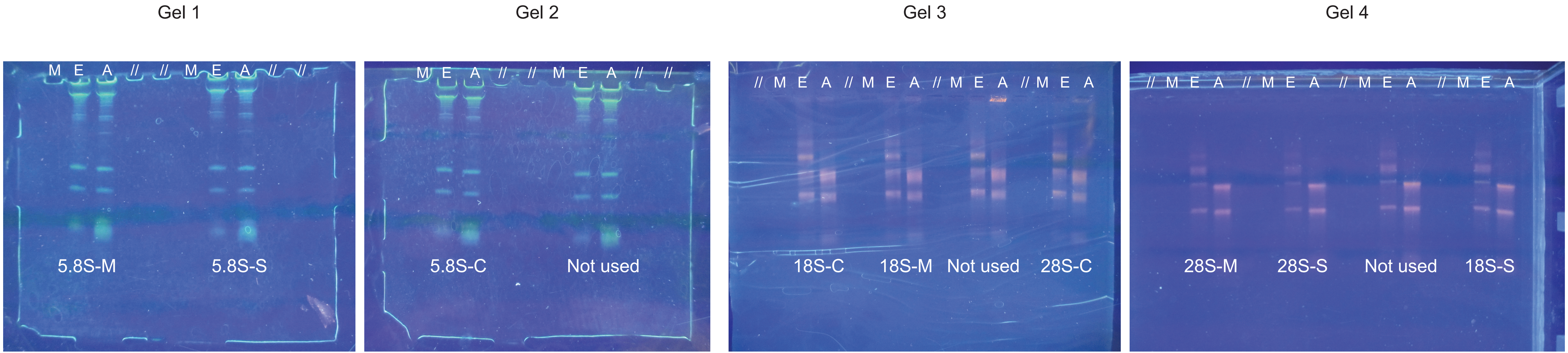

Supplementary Figure S4

The electrophoresis gels that were used in the Northern blots showed in Figure 1C. M= marker, E= egg; A= adult-male whole-body; //= no RNA.
