# Supplementary material for: Expression of distinct maternal and somatic 5.8S, 18S, and 28S rRNA types during zebrafish development
Source: RNA. 2017 Aug;23(8):1188–99. doi: 10.1261/rna.061515.117 (PMC5513064; doi:10.1261/rna.061515.117)
Supplement: Supplemental Material [file supp_061515.117_Supplemental_File_S1.pdf]

Supplementary File S1: Sequence comparison of the 18S (pp. 2-3), 5.8S (p. 4) and 28S (pp. 5-8) rRNA variant types coming from all the genomic 45S rDNA units (Supplementary Table 3A). Nucleotides identical to the first row (45-M) are indicated as dots, while gaps are expressed as dashes.

18S

```
                20        40        60        80        100       120
45S-M 18S  TACCTGGTTGATCCTGCCAGTAATATATGCTTGTCTCAAAGATTAAGCCATGCAAGTCTAAGTGCACACGGCCGGTACAGTGAAACTGCGAATGGCTCATTAAATCAGTTATGGTTCCTTTGATCGCTCC 130
45S-U1 18S .......................................................................A.......................................................... 130
45S-U2 18S .......................................................................A.......................................................... 130
45S-U3 18S .......................................................................A.......................................................... 130
45S-S 18S  .......................C..............................G........................................................................... 130
                140       160       180       200       220       240       260
45S-M 18S  ACCCGGTTACTTGGATAACTGTGGCAATTCCAGAGCTAATACATGCCAACGAGCGCCGACCTGGGCCGCCCTTCTCCCCTCG-G-GGCGGGG-GG--TGGGTACCCGGGGACGCGTGCATTTATCAGATC 255
45S-U1 18S T..............................................................................CTAT.T.......T..GG.....C........................... 260
45S-U2 18S T..............................................................................CTAT.T.......T..GG.....C..T........................ 260
45S-U3 18S T..............................................................................CTAT.T.......T..GG.....C.........................C. 260
45S-S 18S  .T...-.......................................................C..-------.-.-....---------.....-.---------..-........................ 231
                280       300       320       340       360       380
45S-M 18S  CAAAACCCATGCGGGTGCGCGGGCGGTGGAGAGGGGGGCCTCGCGCCTACCCGCCGCCGTCCGCCCCCGGCCTCGCTTTGGTGACTCTAGATAACCTCGGGCCGATCGCGCGCCCTC-GCGGCGGCGACG 384
45S-U1 18S .............................................................................................................A.......-............ 389
45S-U2 18S .......................................T.....................................................................A.......-............ 389
45S-U3 18S ....................................A.....A..................................................................A.......-............ 389
45S-S 18S  ..........C........-.--..-...-.CTCCCC-.....G.GG.-.GG...-T.....-...--....--...........................................C............ 348
                400       420       440       460       480       500       520
45S-M 18S  GTTCATTCGAATGTCTGCCCTATCAACTTTCGATGGTAGGTCCGTCGCCTACCATGGTGACCACGGGTGACGGGGAATCAGGGTTCGATTCCGGAGAGGGAGCCTGAGAAACGGCTACCACATCCAAGGA 514
45S-U1 18S .................................................................................................................................. 519
45S-U2 18S .................................................................................................................................. 519
45S-U3 18S .................................................................................................................................. 519
45S-S 18S  A.....................................CT.TA.G.......................A............................................................. 478
                540       560       580       600       620       640
45S-M 18S  AGGCAGCAGGCGCGCAAATTACCCATTTCCGACACGGAGAGGTAGTGACGAAAAATAACAATGCAGGTCTCTTTCGAGGCCCTGCAATTGGAATGAGTGCATCCCAAACCCATGGGCGAGGACCCATTGG 644
45S-U1 18S .......................................................................C............T............C.T....T......................... 649
45S-U2 18S .......................................................................C............T............C.T....T......................... 649
45S-U3 18S .......................................................................C............T............C.T....T......................... 649
45S-S 18S  ...........................C.........G........................A.....................T............C.T....T......................... 608
                660       680       700       720       740       760       780
45S-M 18S  AGGGCAAGTCTGGTGCCAGCAGCCGCGGTAATTCCAGCTCCAATAGCGTATGCTAACGTTGCTGCAGTTAAAAAGCTCGTAGTTGGATCTCGGGGACCGGGCCGCGCGGTCCGCCGCGAGGCGAGCCACC 774
45S-U1 18S ....................................................................................................................T............. 779
45S-U2 18S ....................................................................................................................T............. 779
45S-U3 18S ....................................................................................................................T............. 779
45S-S 18S  ...................................................AT...A.....................................AGT-....T.-......................... 736
                800       820       840       860       880       900
45S-M 18S  GCCGGTCCCGGACCCCCAGGCCTCCCGGCGCCCCCCGGATGCCCTTGACTGGGTGTCCT-CGGCTTGGGGCCCGGAGCGTTTACTTTGAAAAAATTAGAGTGTTCAAGGCAGGGCCGGCACCGCGCCCCA 903
45S-U1 18S ...........................................................-.........................................................--...T......TT 906
45S-U2 18S ...........................................................-.........................................................--...T......TT 906
45S-U3 18S ...........................................................-.....................................................T....--...T......TT 906
45S-S 18S  ...T...........T--.-..........................AG..........GGTAC..C.........................................A....-....--C..AGC.G..G 860
                920       940       960       980       1,000     1,020     1,040
45S-M 18S  TTGAATACCCCAGCTAGGAATAATGGAATAGGACCCCGGTTCTATTTTCTGTGGGTTTCCGGAACCCGGGGCCATGATCGAGAGGGACGGCCGGGGGCATTCGTATTGCGCCGCTAGAGGTGAAATTCTT 1033
45S-U1 18S A.............................................................................T................................................... 1036
45S-U2 18S A.............................................................................T................................................... 1036
45S-U3 18S A.............................................................................T................................................... 1036
45S-S 18S  C........G........................T.............--.........T..................T................................................... 988
                1,060     1,080     1,100     1,120     1,140     1,160
45S-M 18S  GGACCGGCGCAAGACGGACCGGAGCGAAAGCGTTTGCCAAGAACGTTTTCATTAATCAAGAACGAAAGTCGGAGGTTCGAAGACGATCAGATACCGTCGTAGTTCCGACCGTAAACGATGCCGACCCGCG 1163
45S-U1 18S ...................T.............................................................................................................. 1166
45S-U2 18S ...................T.............................................................................................................. 1166
45S-U3 18S .................................................................................................................................. 1166
45S-S 18S  ...................GAA.........A...........T...................................................................................... 1118
```

18S

```
                     1,180               1,200               1,220               1,240               1,260               1,280               1,300
45S-M 18S  ATCCGGCGGCGTTTATTCC-CA-TGACCCGCCGGGCAGCGTTGCGGGAAACCACGAGTCTCTGGGCTCCGGGGGGAGTATGGTTGCAAAGCTGAAACTTAAAGGAATTGACGGAAGGGCACCACCAGGAG 1291
45S-U1 18S .............AC.A..A..A..................-.T........................T............................................................. 1295
45S-U2 18S .............AC.A..A..A..................-.T........................T............................................................. 1295
45S-U3 18S .............AC.A..A..A..................-.T........................T............................................................. 1295
45S-S 18S  .............AT.-..-..-..................-.........T.............T................................................................ 1244
                               1,320               1,340               1,360               1,380               1,400               1,420
45S-M 18S  TGGAGCCTGCGGCTTAATTTGACTCAACACGGGGAACCTCACCCGGCCCGGACACGGAAAGGATTGACAGATTGACGGCTCTTTCTCGATTCTGTGGGTGGTGGTGCATGGCCGTTCGTAGTTGGTGGAG 1421
45S-U1 18S ...........................................................................TA..........................................G.......... 1425
45S-U2 18S ...........................................................................TA..........................................G.......... 1425
45S-U3 18S .................................................A.........................TA..........................................G.......... 1425
45S-S 18S  .................................A.........................................TA........................................T............ 1374
                     1,440               1,460               1,480               1,500               1,520               1,540               1,560
45S-M 18S  CGATTTGTCTGGTTGATTCCGATAACGAACGAGACTCTGGCATGCTAACTAGTTACGCGGCCCCGCGCGGTCGGCGTCTGCAACTTCTTAGAGGGACAAGTGGCGTTCAGCCACGCGAGACTGAGCAATA 1551
45S-U1 18S T.......................................................................A......................................................... 1555
45S-U2 18S T.......................................................................A......................................................... 1555
45S-U3 18S T.......................................................................A......................................................... 1555
45S-S 18S  ..............C......................C..........A.............................C-........................................TG........ 1503
                               1,580               1,600               1,620               1,640               1,660               1,680
45S-M 18S  ACAGGTCTGTGATGCCCTTAGATGTCCGGGGCTGCACGCGCGCCACAATGGGCGGATCAACGTGTGCCTACCCTGCGCCGACAGGCGCGGGTAACCCGTTGAACCCCGCCCGTGATGGGGACCGGGGATT 1681
45S-U1 18S .................................................................................G.....................A.....T......T............. 1685
45S-U2 18S .................................................................................G.....................A.....T......T............. 1685
45S-U3 18S .................................................................................G.....................A.....T......T............. 1685
45S-S 18S  .................................................................................G...........................T......T.....T....C.. 1633
                     1,700               1,720               1,740               1,760               1,780               1,800               1,820
45S-M 18S  GAAACTATTTCCCGAGAACGAGGAATTCCCAGTAAGCGCAGGTCATCAGCTTGCGTTGATTAAGTCCCTGCCCTTTGTACACACCGCCCGTCGCTACTACCGATTGAGCGGCTCAGTGAGGTCCTCGGAT 1811
45S-U1 18S .C...........TT........................T..........CA...C........................................................A................. 1815
45S-U2 18S .C...........TT........................T..........CA...C........................................................A................. 1815
45S-U3 18S .C...........TT........................T..........CA...C........................................................A................. 1815
45S-S 18S  ......G......ATC..............................A...........................................................GAT..T.T................ 1763
                               1,840               1,860               1,880               1,900               1,920               1,940
45S-M 18S  CGGCCCCGCCCGGGGCTCCCTTACCGGGGGCCCTGGTGGAGCGCCGAGAAGACGATCGAACTCGGTCGTTTAGAGGAAGTAAAAGTCGTAACAAGGTTTCCGTAGGTGAACCTGCGGAAGGATCATTA 1939
45S-U1 18S ................-..T...T..A.--.........TA.A....................T................................................................ 1940
45S-U2 18S ................-..T...T..A.--.........TA.A....................T................................................................ 1940
45S-U3 18S ................-..T...T..A.--.........TA...T..................T................................................................ 1940
45S-S 18S  ..........-.....-T...CG.....--......C....................A....T.A..C.C.......................................................... 1887
```

5.8S

```
                                20                  40                  60                  80                  100                 120
45S-M 5.8S   -AACTCTTAGCGGTGGATCACTCGGCTCGTGCGTCGATGAAGAACGCAGCTAGCTGCGAGAACTAATGTGAATTGCAGGACACACATTGATCATCGACCTTTCGAACGCACATTGCGGCCCCGGGTCCAT 129
45S-U1 5.8S  C..............................................................................--......C..........AC...........T..............T.C. 128
45S-U2 5.8S  C..............................................................................--......C..........AC...........T..............T.C. 128
45S-U3 5.8S  C..............................................................................--......C..........AC...........T..............T.C. 128
45S-S 5.8S   C..............................................................................--......C..........AC...........T..............T.C. 128

                       140
45S-M 5.8S   CCCGGGGCCACGCCTGTCTGAGGGTCGCC 158
45S-U1 5.8S  ......................C.....T 157
45S-U2 5.8S  ......................C.....T 157
45S-U3 5.8S  T.....................C.....T 157
45S-S 5.8S   ......................C.....T 157
```

28S

```
                      20                          40                          60                          80                          100                         120
45S-M 28S  CACGACCTCAGCTCAGACGAGAAGACCCGCTGAATTTAAGCATATTACTAAGCGGAGGAAAAGAAACCAACCGGGATTCCCCCAGTAGCGGCGAGCGAAGAGGGAAAAGTCCAGCGCCGAATCCCCGCCC 130
45S-U1 28S T . . . . . . . . . A . . . . . . . . . CA . . . . . . . . . . . . . . . . . . . . . . . . . . . . . . . . . . . . . . . T . . . A . . . . . . . T . . . . . . . . . . . . . . . . . . . . . . G . . C . . . . . . . . . . . . . . . . . . . 130
45S-U2 28S T . . . . . . . . . A . . . . . . . . . CA . . . . . . . . . . . . . . . . . . . . . . . . . . . . . . . . . . . . . . . T . . . A . . . . . . . T . . . . . . . . . . . . . . . . . . . . . . G . . C . . . . . . . . . . . . . . . . . . . 130
45S-U3 28S T . . . . . . . . . A . . . . . . . . . CA . . . . . . . . . . . . . . . . . . . . . . . . . . . . . . . . . . . . . . . T . . . A . . . . . . . T . . . . . . . . . . . . . . . . . . . . . . G . . C . . . . . . . . . . . . . . . . . . . 130
45S-S 28S  T . . . . . . . . . A . . . . . . . . . CA . . . . . . . . . . . . . . . . . . . . . . . . . . . . . . . . . . . . . . . T . . . A . . . . . . . T . . . . . . . . . . . . . . . . . . . . . . G . . C . . . . . . . . . . . . . . . . . . . 130

             140                         160                         180                         200                         220                         240                         260
45S-M 28S  CTCTGCCGAGGGCGAGGGACCTGTGGCGTACGGAGGGCCGCCTCTCTCG--GCGCGGGCCGGGGGGCCAAAGTCCTTCTGATGGAGGCTTAGCCCGCGGACGGTGTGAGGCCGGTGTCGGCCCCC-GCCC 257
45S-U1 28S - . G . . A . AG . . . T . . . . . AA . . . . . . . . . . . . A . AT . . . TCTCTCTCTC . GCGC . . G . C . . . . . T . T . . . . . . . . . . . . . . . . . . . . . . . . . . T . . . . . . . . . . . . . . . . . AA . . . . . . . T . . . . 259
45S-U2 28S - . G . . A . AG . . . T . . . . . AA . . . . . . . . . . . . A . AT . . . TCTCTCTCTC . GCGC . . G . C . . . . . T . T . . . . . . . . . . . . . . . . . . . . . . . . . . T . . . . . . . . . . . . . . . . . AA . . . . . . . T . . . . 259
45S-U3 28S - . G . . A . AG . . . T . . . . . AA . . . . . . . . . . . . A . AT . . . TCTCTCTCTC . GCGC . . G . C . . . . . T . T . . . . . . . . . . . . . . . . . . . . . . . . . . T . . . . . . . . . . . . . . . . . AA . . . . . . . T . . . . 259
45S-S 28S  - CG . . . . AG . . . . . . . . . AA . . . . . . . . . . . . A . AT . . . TCTCTCTC- - . GCGC . . G . C . . . . . T . T . . . . . . . . . . . . . . . . . . . . . . . . . . T . . . . . . . . . . . . . . . . . AA . . . . . . . - . . . . 256

                      280                         300                         320                         340                         360                         380
45S-M 28S  CGCCGGGGTGCGGTTCCTCCCGGAGTCGGGTTGTTTGGGAATGCAGCCCAAAGCGGGTGGTAAACTCCATCTAAGGCTAAATACCGGCACGAGACCGATAGCGGACAAGTACCGTGAGGGAAAGTTGAAA 387
45S-U1 28S . . . . . . . . A . . . - . T . . . . . . . . . . . . . . . . . . . . . . . . . . . . . . . . . . . . . . . . . . . . . . . . . . . . . . . . . . . . . . . . . . . . . . . . . . . . T . . . . . . . . . . A . . . . . . . . . . . . . . . . . . . . . . . . 388
45S-U2 28S . . . . . . . . A . . . - . T . . . . . . . . . . . . . . . . . . . . . . . . . . . . . . . . . . . . . . . . . . . . . . . . . . . . . . . . . . . . . . . . . . . . . . . . . . . . . . . . . . . . . . . . . A . . . . . . . . . . . . . . . . . . . . . . . . 388
45S-U3 28S . . . . . . . . A . . . - . T . . . . . . . . . . . . . . . . . . . . . . . . . . . . . . . . . . . . . . . . . . . . . . . . . . . . . . . . . . . . . . . . . . . . . . . . . . . . . . . . . . . . . . . . . A . . . . . . . . . . . . . . . . . . . . . . . . 388
45S-S 28S  . . . . . . . . A . . . - . T . . . . . . . . . . . . . . . . . . . . . . . . . . . . . . . . . . . . . . . . . . . . . . . . . . . . . . . . . . . . . . . . . . . . . . . . . . . . . . . . . . . . . . . TC . . . . . . . . . . . . . . . . . . . . . . . . . 385

             400                         420                         440                         460                         480                         500                         520
45S-M 28S  AGAACTTTGAAGAGAGAGTTCAACAGGGCGTGAAACCGTTAAGAGGTAAACGGGTGGGGACCGCACCGTCCGCCCGGTGGATTCAGCCCGGCGGGGCGGGGTCGGCCCGTCCGGTGCGCGCTCCCTTCGC 517
45S-U1 28S . . . . . . CAG . TT . . . . . . A . . . . . . . . . . . . . . . . . . . . . . . . A . . . . . . . . . . . CT . . . . . G . G . T . . . . . A . . . . . . . . . T . . . TT . T . . . - . A . . . T . . . . . . - . - . A . . . TGG- . T- - 512
45S-U2 28S . . . . . . CAG . TT . . . . . . A . . . . . . . . . . . . . . . . . . . . . . . . . . . . . . . . . . . . . . CT . . . . . G . G . T . . . . . A . . . . . . . . . T . . . TT . T . . . - . A . . . T . . . . . . - . - . A . . . TGG- . T- - 512
45S-U3 28S . . . . . . CAG . TT . . . . . . A . . . . . . . . . . . . . . . . . . . . . . . . . . . . . . . . . . . . . . CT . . . . . G . G . T . . . . . A . . . . . . . . . T . . . TT . T . . . - . A . . . T . . . . . . - . - . A . . . TGG- . T- - 512
45S-S 28S  . . . . . . . . . . . . . . . . . . . . . . . . . . . . . . . . . . . . . G . . . . . . . . . . . . . . . . . T . . . . . G . . . . . . . . . A . . . . . . A . . . . . . . . . . . T . . - . . . . . . . . . . . . . . - T- . . . . . . GGA . . - . 510

                      540                         560                         580                         600                         620                         640
45S-M 28S  TCCCTCATTCCTGGGGGTGGCGCGGGGGGTTGACGCCCGGGCGAAGGCTCGGCCGCCGCCGGGTGCATTTCCGCCG-CGGTGGAGCGCCGCGACCGGCTCCGGTTCGGCTTGGAAGGGTCAGGGGGCGAA 646
45S-U1 28S . . - . . - . - T- . - . . . . . - - A- . - A . . . A- CC . G . . . . . . . . . AG . . - T . . T . . . . G . . T . T . . . . . . C . . T . T . . . T . . . C . . T . . . . . . . . . . . . . . . . . . . . . . . . . . . . . . . . . TC . . . AA . . . . 631
45S-U2 28S . . - . . - . - T- . - . . . . . - - A- . - A . . . A- CC . G . . . . . . . . . AG . . - T . . T . . . . G . . T . T . . . . . . C . . T . T . . . T . . . C . . T . . . . . . . . . . . . . . . . . . . . . . . . . . . . . . . . . TC . . . AA . . . . 631
45S-U3 28S . . - . . - . - T- . - . . . . . - - A- . - A . . . A- CC . G . . . . . . . . . AG . . - T . . T . . . . G . . T . T . . . . . . C . . T . T . . . T . . . C . . T . . . . . . . . . . . . . . . . . . . . . . . . . . . . . . . . . TC . . . AA . . . . 631
45S-S 28S  C . . - C . - CCT . . C . . . . - . A- . - . . . . A- CC . G . . . . . . . A . . G . . - . . . . . . . . . . . . . . . . C . . . C . . . . T . . . T . . . C . . T . . . . . . . . . . . . . . . . . . . . . . . . . . . . . . . . . TC . . . . . . . . 633

             660                         680                         700                         720                         740                         760                         780
45S-M 28S  GGTGGCCCGTCGGTTCAGGCCGTCGGGCTTTACAGCGCCCTCCCGCCCCGACTTCGCCGCTTGCTCTCCGGGGCCGCGGGTGAGTGTCCTCCGCGCCCTCTCTGCCCTCCCTCCCTGTGGGGGGAGGTGC 776
45S-U1 28S . . . . . T . . . - . . C . . C . . . . A . - . A . . . . . . . . . . . . T . . C . T- . . . . . C . . . . . . . . . . . . . . . AC . . . . . . . . T . . . C- . . . . AT . . . . . . . . . . . . . . . C . G- . . - - . - TG . CG . . A- - - - C- - . G 747
45S-U2 28S . . . . . T . . . - . . C . . C . . . . A . - . A . . . . . . . . . . . . T . . C . T- . . . . . C . . . . . . . . . . . . . . . AC . . . . . . . . T . . . C- . . . . AT . . . . . . . . . . . . . . . C . G- . . - - . - TG . CG . . A- - - - C- - . G 747
45S-U3 28S . . . . . T . . . - . . C . . CA . . . A . - . A . . . . . . . . . . . . T . . C . T- . . . . . C . . . . . . . . . . . . . . . AC . . . . . . . . T . . . C- . . . . AT . . . T . . . . . . . . . . . C . G- . . - - . - TG . CG . . A- - - - C- - . G 747
45S-S 28S  . . . . . T . . C- . . C . . C . . . TC- . A . . . . . . . . . . . . . . C . . . . . . . . . . . . . . . . . . . . . . . . . . AC . . . . . . . . . . . . . C- . . . . . . . . . . . . . . . T . . . . . C . GGC . . . . . T . . CG . . . . . GCTC . G 760

                      800                         820                         840                         860                         880                         900
45S-M 28S  GGGGACGGG-GTCCCC-CGCCCCCGGCGTGGCGCGACAGGGGTGGACTGTCCTCAGTCCGCCCACGGCTGCGCCGCGCCGCCCAGGGCGGGGA-T--CCGACCCACGTTCGGGCGCCCGAGGTCCGCGGC 901
45S-U1 28S - . . - - . T- - - C . T . G . - . C . . . GGC . . . . . C . T . AG . C . . ATC . . . . . . . T . . . . . . . TCTATC . . . T . . T . . . . T . . T . . T . G . . . T . . . . G- A- - - - - . - G- - - A- - - . . . . . . . . . T . . . . . . . . . T 857
45S-U2 28S - . . - - . T- - - C . T . G . - . C . . . GGC . . . . . C . T . AG . C . . ATC . . . . . . . T . . . . . . . TCTATC . . . T . . T . . . . T . . T . . T . G . . . T . . . . G- A- - - - - . - G- - - A- - - . . . . . . . . . T . . . . . . . . . T 857
45S-U3 28S - . . - - . T- - - C . T . G . - . C . . . GGC . . . . . C . T . AG . C . . ATC . . . . . . . T . . . . . . . TCTATC . . . T . . T . . . . T . . T . . T . G . . . T . . . . G- A- - - - - . - G- - - A- - - . . . . . . . . . T . . . . . . . . . T 857
45S-S 28S  A . . . . . . . . GCC . . T . G . T . . . GGC . . . . . CGT . . . . C . . AAC . . . . . . . . . . . . . . . . . . . . TTTC . . A . C . . . . . . T . . . . . . . . . . . . . . . CAGG . . C . . GTC . AAAA . . . . . . . . . . . . . . . . . . T 890

             920                         940                         960                         980                         1,000                       1,020                       1,040
45S-M 28S  GACGCCGGCCTCCCACCCGACCCGTCTTGAAACACGGACCAAGGAGTCCAACGCGCGCGCGAGTCAGAGGGTGGCTCGCGAGCCCCTGCGGCGCAATGAAGGTGAGAACGGGGGTCT-CC--CCCGGGTG 1028
45S-U1 28S . . T . . . . . . . A . . . . . . . . . . . . . . . . . . . . . . . . . . . . . . . . . . . . . . . . . . . . . . . . . . . . . . A . . . . A . . . . . . - TC . T . . . . . . . . CA . . . . . . . . . . . . . . . . . AGG . C . . C . . G . G . . GG . . . A . . . . 986
45S-U2 28S . . T . . . . . . . A . . . . . . . . . . . . . . . . . . . . . . . . . . . . . . . . . . . . . . . . . . . . . . . . . . . . . . A . . . . A . . . . . . - TC . T . . . . . . . . CA . . . . . . . . . . . . . . . . . AGG . C . . C . . G . G . . GG . . . A . . . . 986
45S-U3 28S . . T . . . . . . . A . . . . . . . . . . . . . . . . . . . . . . . . . . . . . . . . . . . . . . . . . . . . . . . . . . . . . . A . . . . A . . . . . . - TC . T . . . . . . . . CA . . . . . . . . . . . . . . . . . AGG . C . . C . CG . G . . GG . . . A . . . . 986
45S-S 28S  . . T . . . . . . . A . . . . . . . . . . . . . . . . . . . . . . . . . . . . . . . . . . . . T . . . . . . . . . . . . . . . . . . . . . . . . . - . C . T . . . . . . . . CA . . . . . . . . . . . . . . . . . AGG . C . . C . CGCG . . GG . . . A . . . . 1019

                      1,060                       1,080                       1,100                       1,120                       1,140                       1,160
45S-M 28S  GGATCCCCCCGCCCCGGCGGGGGGCGCACCACCGGCCCGCCTCGCGACCCTCCGGGGGGCAGGTGGAGTTGGAGCGCGCGCGATGGCACCCGAAAGATGGTGAACTATGCCTGGGCAGGGCGAAGCCAGG 1158
45S-U1 28S . . . . . . . . . . . TT . . . . . . . . . . . . . . . . . . . . . . . . . . . . T . . . . . C- . G . . . . . CC . . . G . . . . . . . . CAA . . . . . T . . . . . . . . . T . . . . . . . . . . . . . . . . . . . . . . . . . . . . . . . . . . T . . . . . . . . 1115
45S-U2 28S . . . . . . . . . . . TT . . . . . . . . . . . . . . . . . . . . . . . . . . . . T . . . . . C- . G . . . . . CC . . . G . . . . . . . . CAA . . . . . T . . . . . . . . . T . . . . . . . . . . . . . . . . . . . . . . . . . . . . . . . . . . T . . . . . . . . 1115
45S-U3 28S . . . . . . . . . . . TT . . . . . . . . . . . . . . . . . . . . . . . . . . . . T . . . . . C- . G . . . . . CC . . . G . . . . . . . . CAA . . . . . T . . . . . . . . . T . . . . . . . . . . . . . . . . . . . . . . . . . . . . . . . . . . T . . . . . . . . 1115
45S-S 28S  . . . . . . . . . . . . . . . . . . . . . . . . . . . . . . . . . . . . . . . . T . . . . TC- . G . . . T . TC . . . G . . . . . . . . C . A . . . . . . . T . . . . . . . . . T . . . . . . . . . . . . . . . . . . . . . . . . . . . . . . . . . . . . . . . . . . . . A 1148
```

28S

```
          1,180               1,200               1,220               1,240               1,260               1,280               1,300
45S-M 28S  GGAAACTCTGGTGGAGGCCCGCCGCGGTCCTGACGTGCAAATCGGTCGTCCGACCTGGGCATAGGGGCGAAAGACTAATCGAACCATCTAGTAGCTGGTTCCCTCCGAAGTTTCCCTCAGGATAGCTGGC 1288
45S-U1 28S .....................TA....................................T..............................A....................................... 1245
45S-U2 28S .....................TA....................................T..............................A....................................... 1245
45S-U3 28S .....................TA....................................T..............................A....................................... 1245
45S-S 28S  ......................A....................................T...................................................................... 1278

          1,320               1,340               1,360               1,380               1,400               1,420
45S-M 28S  GCTCGCCGATCAAGCAGTTTTATCCGGTAAAGCCAATGACTAGAGGCCTTGGGGCCGAAACGGCCTCAACCTATTCTCAAACTTTAAATGGGTAAGAGGCCCGGCTCGCTGGCGCTGGAGCCGGGCGTGG 1418
45S-U1 28S .....AT-.-.--....................G...............................................................A...........A...-T............... 1370
45S-U2 28S .....AT-.-.--....................G...............................................................A...........A...-T............... 1370
45S-U3 28S .....AT-.-.--....................G...............................................................A...........A...-T............... 1370
45S-S 28S  .......-.-.--....................G............................A..................................A...............-T............... 1403

          1,440               1,460               1,480               1,500               1,520               1,540               1,560
45S-M 28S  AATGCGACGCGCCTAGTGGGCCATTTTTGGTAAGCAGAACTGGTGCTGCGGGATGAACCGAACGCCGGGTTAAGGCGCCCGATGCCGACGCTCATCAGACCCCATAAAAGGTGTTGGTTGATATAGACAG 1548
45S-U1 28S .......GA....C........G....................C.....................T................................................................ 1500
45S-U2 28S .......GA....C........G....................C.....................T................................................................ 1500
45S-U3 28S .......GA....C........G....................C.....................T................................................................ 1500
45S-S 28S  .......GA....C........T....................C............................................................G......................... 1533

          1,580               1,600               1,620               1,640               1,660               1,680
45S-M 28S  CAGGACGGTGGCCATGGAAGTCGGCACCCGCCAAGGAGTGTGTAACAACTCACCTGCCGAATCAACTAGCCCTGAAAATGGATGGCGCTGGAGCGTCGGGCCCATACCCGGCCGTCGACGGCACAAGGGA 1678
45S-U1 28S ...............................T...........................................................................................G....AG 1630
45S-U2 28S ...............................T...........................................................................................G....AG 1630
45S-U3 28S ...............................T.............................................................G.............................G....AG 1630
45S-S 28S  ........................A.T....T...........................................................................................G....AG 1663

          1,700               1,720               1,740               1,760               1,780               1,800               1,820
45S-M 28S  CA-CGCGAGCGGTCGCGC-GCGCTGCAAGCCTCGACGAGTAGGAGGGCCGCCGCGGTGGCGCCGAAGCCCAGGGCGCGGGCCCGGGTGGAGCCGCCGCGGGCGCAGATCTTGGTGGTAGTAGCAAATATT 1806
45S-U1 28S .CC.C.CTC.C.GG.G.G-AAAGG.T....................................G......T......T...............A........T............................ 1759
45S-U2 28S .CC.C.CTC.C.GG.G.G-AAAGG.T....................................G......T......T...............A........T............................ 1759
45S-U3 28S .CT.C.CTC.C.GG.G.G-AAAGG.T....................................G......T......T...............A........T............................ 1759
45S-S 28S  .CC.C.CCT.C.GG.G.GGAAAGG.T....................................G......T......T........................T............................ 1793

          1,840               1,860               1,880               1,900               1,920               1,940
45S-M 28S  CAAACGAGAGCTTTGAAGGCCGAAGTGGAGAAGGGTTCCATGTGAACAGCAGTTGAACATGGGTGAGCCGGTCCTAAGGGACGGGCCTACGCCGTTCGGAGGGGAGGGGCGATGGCCTCTGTCGCCCCCG 1936
45S-U1 28S ....................................................A..............T.............T....GA....T.......A..................C.......... 1889
45S-U2 28S ....................................................A..............T.............T....GA....T.......A..................C.......... 1889
45S-U3 28S ....................................................A..............T.............T....GA....T.......A.....A............C.......... 1889
45S-S 28S  ................................................................C..T.............T....GA............A......A...........C.......... 1923

          1,960               1,980               2,000               2,020               2,040               2,060               2,080
45S-M 28S  CTCGACCGAAAGGGAGTCGGGTCCAGATCCCCGAGCCCGGAGCGGCGGAGACGGGCGCCGCGAGGCGCCCAGTGCGGTGACGCAAACGAACCCGGAGATGCCGGCGGGTGCCCCGGGAAAGAGTTCTCTT 2066
45S-U1 28S GC...T................T...........A.......T.......................................................A.......T.A....T.....-.......... 2018
45S-U2 28S GC...T................T...........A.......T.......................................................A.......T.A....T.....-.......... 2018
45S-U3 28S GC...T................T...........A.......T.......................................................A.......T.A..........-.......... 2018
45S-S 28S  GC...T................T...........A.......T.......................................................A.........A..........-.......... 2052

          2,100               2,120               2,140               2,160               2,180               2,200
45S-M 28S  TTCTTTGTGAAGGGCAGGGCGCCCTGGAACGGGTTCGCCCCGAGAGAGGGGCCCGCGCCCTGGAAAGCGCCGCGCTTCTGGCGGCGTCCGGTGAGCTCTCGTCGGCCCTTGAAAATCCGGGGGAGAAGGT 2196
45S-U1 28S .............................T......AG..................................T.A..GC...........................................A....... 2148
45S-U2 28S .............................T......AG..................................T.A..GC...........................................A....... 2148
45S-U3 28S .............................T......AG..................................T.A..GC...........................................A....... 2148
45S-S 28S  .............................T............................................G...C................................................... 2182

          2,220               2,240               2,260               2,280               2,300               2,320               2,340
45S-M 28S  GTAAATCTCGCGCCGGGCCGTACCCATATCCGCAGCAGGTCTCCAAGGTGAACAGCCTCTGGCGTGTTGGAACAAGGCAGAGTAAGGGAAGTCGGCAAGTCAGATCCGTAACTTCGGGATAAGGATTGGC 2326
45S-U1 28S ................................................................................-................................................. 2277
45S-U2 28S ................................................................................-................................................. 2277
45S-U3 28S ....G.........................................................................G.-................................................. 2277
45S-S 28S  ....G...........................................................................-................................................. 2311
```

28S

```
                        2,360               2,380               2,400               2,420               2,440               2,460
45S-M 28S   TCTAAGGGCTGAGCCGGTCGGGCTGAGGTGCGAAGCGGGCCTGGGCCCGAGCCGCGACTGGGGAGCGGCCGCCCCGAGGTGCCCTGACCCCCGTTCCCGAGCCGCGGGGTGCGCGCGCGGTGGCGCGC 2456
45S-U1 28S  ..........G..................A........G.......A.T.T....G..................TT.......CCCTG.AT.......T-TT-.------..TA-..A..-.---.-..T.. 2396
45S-U2 28S  ..........G..................A........G.......A.T.T....G..................TT.......CCCTG.AT.......T-TT-.------..TA-..A..-.---.-..T.. 2396
45S-U3 28S  ..........G..................A........G.......A.T.T....G..................TT.......CCCTG.AT.......T-TT-.------..TA-..A..-.---.-..T.. 2396
45S-S 28S   ..........G...........................G.........C......G...................C..C....CC-T-.T.........T-.T-.-----C.GA-........-.---.-..T.. 2428

                 2,480               2,500               2,520               2,540               2,560               2,580               2,600
45S-M 28S   GGTCCCTCTCTCCCCCCGCCGCGTCCGCCTCGGGCCTTTTTGCGGTCTCGACTCCCCCGACTCTTCCCCTCCGCCCTTTCCCCGTCCGCGGGGTCGGCGGGGGGGTCTCGGAGGAGGGGGGGTGGAG 2586
45S-U1 28S  ..-...-..AG.....T-.-.--.-.-.-...-..-.-...C..-..ATG.G..-...G..G....A-..GA---.AA..C-........T..CT--.......A..C...GG--..G..C..AT.TC..T-. 2501
45S-U2 28S  ..-...-..AG.....T-.-.--.-.-.-...-..-.-...C..-..ATG.G..-...G..G....A-..GA---.AA..C-........T..CT--.......A..C...GG--..G..C..AT.TC..T-. 2501
45S-U3 28S  ..-...-..AG.....T-.-.--.-.-.-...-..-.-...C..-..ACG.G..-...G..G....A-..GA---.AA..C-........T..CT--.......A..C...GA--...A-C..AT.TC..T-. 2500
45S-S 28S   ..-...-..A-....-..-.--.-.-.-...-..-.-...C..-...T-.G..-...G-----.----.G-------C-G...C-..CT--.......T-..-...A-..---C---C------. 2503

                        2,620               2,640               2,660               2,680               2,700               2,720
45S-M 28S   AGGCCGTGGGGAGGAAAGGGGTGGTTCGTGGCGTCGGGGGGCAACGGGAGGGTCCTCGCCTGCCGCGCGATGCCTCCGCTGGCCGGGGCCCGCGGGGGGCGGATGCGCAGCGGTTGGCGGCGGCGACCC 2716
45S-U1 28S  C...T-C.T.T-.CTC-.......G.-..T....-.T...-T--.......-....GTCT.T.-..-..-..G-...AA-.--..A....TGTGTGTA....G.........T....--..T.........TG 2613
45S-U2 28S  C...T-C.T.T-.CTC-.......G.-..T....-.T...-T--.......-....GTCT.T.-..-..-..G-...AA-.--..A....TGTGTGTA....G.........T....--..T.........TG 2613
45S-U3 28S  C...T-C.T.T-.CTC-.......G.-..T....-.T...-T--.......-....GTCT.T.-..-..-..G-...AA-.--..A....TGTGTGTA....G..-.......T....--..T.....T...TG 2611
45S-S 28S   C...T-C.T..-.CTC-.......G.-..T...G-.TA..---------.-..-.GTC....-..-.G-..C-...GA-.--..T.......T........G.ATGC..TGC.GT--T...........T. 2612

                 2,740               2,760               2,780               2,800               2,820               2,840               2,860
45S-M 28S   TGGGCGCGCGCCGCGCCCTTCCCGCGGATCTCCGCAGCTACGGCCCCGCGCCGGGGCCCGCGTCCGCGCGTGCGCCCCCCTCCGGGGGAGTGGCGCGC-GGCCT-CG-GC-TCCCCCCGGTGCGGGCGCC 2842
45S-U1 28S  ...A...A...A.G...G...T..T.....C.TCTG...........-...T....A..TCTCGT.TA...GCTT.T......G....AG.G..TAG.TC.CGGGG..A.TG...T...C.GCGC..T... 2742
45S-U2 28S  ...A...A...A.G...G...T..T.....C.TCTG...........-...T....A..TCTCGT.TA...GCTT.T......G....AG.G..TAG.TC.CGGGG..A.TG...T...C.GCGC..T... 2742
45S-U3 28S  ...A...A...A.G...G...T..T.....C.TCTG...........-...T....A..TCTCGT.TA...ACTT.T......G....AG.G..TAG.TC.CGGGG..A.TG...T...C.GCGC..T... 2740
45S-S 28S   ...A..T......G.......T............C............-...T....A..TC-CGT.TG....CTT.T..T...G....-GAG...A..G-.CGGG-..-.---..T...C.GCGC...... 2733

                        2,880               2,900               2,920               2,940               2,960               2,980
45S-M 28S   TCGGCCGGCGGC-TAGCAGCCAGCTTAGAACTGTCGCGGACCAGGGGAATCCGACTGTTTAATTAAAACAAAGCATCGCGAAGGCCCTCGGCGGGTGTTGACGCGATGTGATTTCTGCCCAGTGCTCTGA 2971
45S-U1 28S  ........TAAGA.T................................................................................................................... 2872
45S-U2 28S  ........TAAGA.T................................................................................................................... 2872
45S-U3 28S  ........TAAGA.T..........................................................................A........................................ 2870
45S-S 28S   ..........C.-.......TG..........GT....................................................GT........................................ 2862

                 3,000               3,020               3,040               3,060               3,080               3,100               3,120
45S-M 28S   ATGTCAAAGTGAAGAAATTCAACGAAGCGCGGGTAAACGGCGGGAGTAACTATGACTCTCTTAAGGTAGCCAAATGCCTCGTCATCTAATTAGTGACGCGCATGAATGGATGAACGAGATTCCCACTGTC 3101
45S-U1 28S  .................................................................................................................................. 3002
45S-U2 28S  .................................................................................................................................. 3002
45S-U3 28S  .................................................................................................................................. 3000
45S-S 28S   ......................T........................................................................................................... 2992

                        3,140               3,160               3,180               3,200               3,220               3,240
45S-M 28S   CCTACCTGCTATCTAGCGAAACCACAGCCAAGGGAACGGGCTTGGCAGAATCAGCGGGGAAAGAAGACCCTGTTGAGCTTGACTCTAGTCTGGCCCTGTGAAGAGACATGAGGGGTGTAGAATAAGTGGG 3231
45S-U1 28S  ..............................................................................................A................................... 3132
45S-U2 28S  ..............................................................................................A................................... 3132
45S-U3 28S  ..............................................................................................A................................... 3130
45S-S 28S   ..............................................................................................A................................... 3122

                 3,260               3,280               3,300               3,320               3,340               3,360               3,380
45S-M 28S   AGGCCCCCGGGCTTCCCGGGCCGGCCGCCGGTGAAATACCACTACTCTTATCGTTTCCTCACTTACCCGGTGAGGCGGGGAAGCCGGGCGTCCCCCGGCGGGGCCGCCCCTGCTTCTGGCGTCAAGCGC 3361
45S-U1 28S  ............A..........................................................T...A...G.TT...T.G.T.TT..TCA.T..TCT...C.........T.......... 3262
45S-U2 28S  ............A..........................................................T...A...G.TT...T.G.T.TT..TCA.T..TCT...C.........T.......... 3262
45S-U3 28S  ............A..........................................................T...A...G.TT...T.G.T.TT..TCA.T..TCT...C.........T.......... 3260
45S-S 28S   ......TG....CCT.G..C..A..G.G..CC.CCGGTGA.A...CAC..-..-..C.TA..GT..C.T.AC.T.CC...TTGG..A...A.G.GAG.CC.C..C.GGCT..T-.................. 3249

                        3,400               3,420               3,440               3,460               3,480               3,500
45S-M 28S   CCCGGGGCCGTGCGGGGGGTGGGGGCAACCCCCCTTCCCCTCCCGGCCGCCGACCGGGGCGCGACCCGCCCCGGGGACAGCGTCAGGTGGGGAGTTTGACTGGGGCGGTACACCTGTCAAACGGTAACGC 3491
45S-U1 28S  ........--A-A.A-----------.--.------.-..------.---.-..-.----.---...T.............................................................. 3356
45S-U2 28S  ........--A-A.A-----------.--.------.-..------.---.-..-.----.---...T.............................................................. 3356
45S-U3 28S  ........--A-A.A-----------.--.------.-..------.---.-..-.----.---...T.............................................................. 3354
45S-S 28S   .T......--C-C.AT-----------.G-.------G-GT.-----CC--.G-.-.-.----.---...T............T.G............................................. 3344
```

28S

```
                3,520               3,540               3,560               3,580               3,600               3,620               3,640
45S-M 28S  AGGTGTCCTAAGGCGAGCTCAGGGGGGACAGAAACCTCCCGTAGAGCAGAAGGGCAAAAGCTCGCTTGATCTTGATTTTCAGTATGAGTACGGACCGCGAAAGCGGGGCCTCACGATCCTTCCGGCTTTT 3621
45S-U1 28S .................................................................................................................................. 3486
45S-U2 28S .................................................................................................................................. 3486
45S-U3 28S .................................................................................................................................. 3484
45S-S 28S  ........................A................CG......................................................T........................T....... 3474

                      3,660               3,680               3,700               3,720               3,740               3,760
45S-M 28S  GGGGTTTTAAGCGGGAGGTGTCAGAAAAGTTACCACAGGGATAACTGGCTTGTGGCGGCCAAGCGTTCATAGCGACGTCGCTTTTTGATCCTTCGATGTCGGCTCTTCCTATCATTGTGAAGCAGAATTC 3751
45S-U1 28S .................................................................................................................................. 3616
45S-U2 28S .................................................................................................................................. 3616
45S-U3 28S .................................................................................................................................. 3614
45S-S 28S  T...........A..................................................................................................................... 3604

                3,780               3,800               3,820               3,840               3,860               3,880               3,900
45S-M 28S  ACCAAGCGTTGGATTGTTCACCCACTAACAGGGAACGTGAGCTGGGTTTAGACCGTCGTGAGACAGGTTAGTTTTACCCTACTGATGTGAGCCGTTGTTGCAATAGTAATCCCGCTCAGTACGAGAGGAA 3881
45S-U1 28S ..........................................................................................A.A..................................... 3746
45S-U2 28S ..........................................................................................A.A..................................... 3746
45S-U3 28S ..........................................................................................A.A..................................... 3744
45S-S 28S  ............................T...........................................................CC.T-...................T................. 3733

                      3,920               3,940               3,960               3,980               4,000               4,020
45S-M 28S  CCGCGGGTTCAGACATTTGGTTCGTGCGCTTGGCTGAGGAGCCACTGGCGCGAAGCCACCATCTGCGGGATTATGACTGAACGCCTCTAAGTCAGAATCCCGCCTAAAAGCAACGATACAGCAGCGCCGT 4011
45S-U1 28S .................................................................T.....................................................T........A. 3876
45S-U2 28S .................................................................T.....................................................T........A. 3876
45S-U3 28S .................................................................T...............................C.....................T.......... 3874
45S-S 28S  ....A................G....T.....................T.......T........T..........................................C.T........C.........C 3863

                4,040               4,060               4,080               4,100               4,120               4,140               4,160
45S-M 28S  CGGATCTCCGATAGGCCCCGGGTAACCGGGAAGCCCCTCTCCGGGGGGGCCCCCGGCGCGGAGAGCCATTCGTGACAGGAACCGGGGGCGCGGCTAGAACGAGCGCCGCCCCCTCTTCCAGTGACGCACC 4141
45S-U1 28S -.-.............................................C................................................................................. 4004
45S-U2 28S -.-.............................................C................................................................................. 4004
45S-U3 28S -.-.............................................C..................................................T.............................. 4002
45S-S 28S  -.-GGGCTTCGATT.G..T...AT.G.C..CC.GGGT..G..CTC..-T.----..T...........GC......G...-......-......C-................-......G..-....... 3981

                      4,180               4,200               4,220               4,240               4,260               4,280
45S-M 28S  GCATGTTTGTGGGGAACCCGGTGCTTAAATGACTCGTAGACGACCTGATTCTGGGTCGGGGTGTCGTGCGTGGCAGAGCAGCTCTGTTGCTGCGATCCATTGAAAGTCAGCCCTCGATCCAAGTTTTGT 4270
45S-U1 28S ....................................................................................CA.C.....................T.............C...TG 4133
45S-U2 28S ....................................................................................CA.C.....................T.............C...TG 4133
45S-U3 28S .....................................................A..............................CA.C.....................T.............C...TG 4131
45S-S 28S  .........................-...............................A....T.....................CA.C......................................... 4109
```
