# Supplementary material for: Expression of distinct maternal and somatic 5.8S, 18S, and 28S rRNA types during zebrafish development
Source: RNA. 2017 Aug;23(8):1188–99. doi: 10.1261/rna.061515.117 (PMC5513064; doi:10.1261/rna.061515.117)
Supplement: Supplemental Material [file supp_061515.117_Supplemental_Legends.docx]

# SUPPLEMENTARY INFORMATION

Supplemental_Fig_S1.pdf: qPCR results

Supplemental_Fig_S2.pdf: The putative secondary structures for maternal- and somatic-type 18S rRNA Domain A and 3’ minor.

Supplemental_Fig_S3.pdf: The distributions of the per gene (non-equal) ratio of maternal/somatic-type 18S ESs nucleotides that are antisense to the 5'UTR of mRNA

Supplemental_Fig_S4.pdf: Gels used in the Northern blotting

Supplemental_File_S1.pdf: Sequence comparison of the 18S, 5.8S and 28S rRNA variant types coming from all the genomic 45S rDNA units

Supplemental_Table_S1.xlsx: 45S rDNA units in the zebrafish genome and comparison to each other and to human

Supplemental_Table_S2.xlsx: Expression of 45S rDNA types

Supplemental_Table_S3.xlsx: Relative and raw read counts for each rRNAs type per analyzed zebrafish sample

Supplemental_Table_S4.xlsx: Differences in the structural domains between maternal- and somatic-type 18S and 28S rRNA

Supplemental_Table_S5.xlsx: Annotation of the maternal- and somatic-type 18S and 28S rRNA

Supplemental_Table_S6.xlsx: Northern blot DNA probes and PCR primers
